# Is Long-Term Heavy Metal Exposure Driving Carriage of Antibiotic Resistance in Environmental Opportunistic Pathogens: A Comprehensive Phenomic and Genomic Assessment Using *Serratia* sp. SRS-8-S-2018

**DOI:** 10.3389/fmicb.2020.01923

**Published:** 2020-08-20

**Authors:** Sherif Gendy, Ashvini Chauhan, Meenakshi Agarwal, Ashish Pathak, Rajesh Singh Rathore, Rajneesh Jaswal

**Affiliations:** ^1^School of Allied Health Sciences, Florida A&M University, Tallahassee, FL, United States; ^2^Environmental Biotechnology Laboratory, School of the Environment, FSH Science Research Center, Florida A&M University, Tallahassee, FL, United States

**Keywords:** *Serratia*, heavy metal resistance, antibiotic resistance, phenomics, genomics

## Abstract

The carriage of both, heavy metal and antibiotic resistance appears to be a common trait in bacterial communities native to long-term contaminated habitats, including the Savannah River Site (SRS). There is widespread soil contamination at the SRS; a United States Department of Energy (DOE) facility with long-term contamination from past industrial and nuclear weapons production activities. To further evaluate the genomic and metabolic traits that underpin metal and antibiotic resistance, a robust mercury (Hg) and uranium (U)-resistant strain- SRS-8-S-2018, was isolated. Minimum inhibitory concentration of this strain revealed resistance to Hg (10 μg/ml) and U (5 mM), the two main heavy metal contaminants at the SRS. Metabolic assessment of strain SRS-8-S-2018 using Biolog metabolic fingerprinting analysis revealed preference for carbohydrate utilization followed by polymers, amino acids, carboxy acids, and esters; this physiological activity diminished when Hg stress was provided at 1 and 3 μg/ml and completely ceased at 5 μg/ml Hg, indicating that continued release of Hg will have negative metabolic impacts to even those microorganisms that possess high resistance ability. Development of antibiotic resistance in strain SRS-8-S-2018 was evaluated at a functional level using phenomics, which confirmed broad resistance against 70.8% of the 48 antibiotics tested. Evolutionary and adaptive traits of strain SRS-8-S-2018 were further assessed using genomics, which revealed the strain to taxonomically affiliate with *Serratia marcescens* species, possessing a genome size of 5,323,630 bp, 5,261 proteins (CDS), 55 genes for transfer RNA (tRNA), and an average G + C content of 59.48. Comparative genomics with closest taxonomic relatives revealed 360 distinct genes in SRS-8-S-2018, with multiple functions related to both, antibiotic and heavy metal resistance, which likely facilitates the strain’s survival in a metalliferous soil habitat. Comparisons drawn between the environmentally isolated *Serratia* SRS-8-S-2018 with 31 other strains revealed a closer functional association with medically relevant isolates suggesting that propensity of environmental *Serratia* isolates in acquiring virulence traits, as a function of long-term exposure to heavy metals, which is facilitating development, recruitment and proliferation of not only metal resistant genes (MRGs) but antibiotic resistant genes (ARGs), which can potentially trigger future bacterial pathogen outbreaks emanating from contaminated environmental habitats.

## Introduction

Heavy metal contamination is a global environmental problem shown to cause both ecological and public health problems (Tchounwou et al., 2012). However, ecosystems with pervasive and long-term heavy metal contamination, such as the Savannah River Site (SRS), located in Aiken, SC, United States, are known to harbor metal-resistant microorganisms, which have acquired genomic features to resist and circumvent metal toxicity (Ye, 2014; Agarwal et al., 2018; Pathak et al., 2019, 2020; Gendy et al., 2020a, b). The two main heavy metal contaminants within the impacted SRS soils include mercury (Hg) and uranium (U) (Sowder et al., 2003). Our ongoing culture-dependent and culture-independent work on the SRS metalliferous soils has demonstrated several different bacterial groups involved in metal cycling processes, such as *Arthrobacter* spp., *Burkholderia* spp., *Bacillus* spp., *Bradyrhizobium* spp., *Pseudomonas* spp., *Lysinibacillus* spp., *Paenibacillus* spp., *Stenotrophomonas* spp., and *Serratia* spp. (Agarwal et al., 2020a, b; Gendy et al., 2020b). Many of these groups have been demonstrated to cycle Hg by activity of their *mer* operon, which consists of regulatory proteins- MerR and MerD, transport proteins- MerP, T and E, as well as protein(s) with reductase activity- MerA (Agarwal et al., 2019; Pathak et al., 2020). In reference to U cycling processes, several different genes have been shown to render U resistance and biomineralization, such as the gene encoding uranium binding complex (UBC), uranium response in *Caulobacter* (*urcA*), phosphatase genes (*phoY; phoK*; and *phoN*) and PIB-type ATPAse (Martinez et al., 2006; Hillson et al., 2007; Nilgiriwala et al., 2008; Nongkhlaw et al., 2012; Yung and Jiao, 2014; Kulkarni et al., 2016; Thorgersen et al., 2017). However, no specific bacterial operon has been shown to be active under U stress to date.

Despite this advantage of microbially based heavy metal bioremediation, studies have also unequivocally shown that bacteria can acquire antibiotic resistance when exposed to long-term contamination, especially with Hg and U (Benyehuda et al., 2003), including our previous work (Agarwal et al., 2019, 2020a,b; Gendy et al., 2020a, b; Pathak et al., 2020). This builds upon significant evidence that exists on the SRS soils which unequivocally shows higher levels of antibiotic resistance in contaminated SRS soils relative to reference soils (Stepanauskas et al., 2006; Wright et al., 2006; Thomas et al., 2020). Furthermore, both, empirical and anecdotal evidence from SRS soils strongly suggest recruitment of antibiotic resistances in many human pathogens that are native to these soils [e.g., *Burkholderia*, *Ralstonia*, *Massilia*, *Acinetobacter*, and *Pseudomonas* (Stepanauskas et al., 2006)]. Despite the fact that many of these SRS native soil bacteria are bioremediative being armed with an arsenal of metal resistance genes (MRGs), but they can also pose public health risks by virtue of their acquired antibiotic resistance(s), via the activity of antibiotic resistant genes (ARGs), thus strongly negating their beneficial properties. Thus, heavy-metal induced development and carriage of ARGs, or the ‘resistome’, within the native soil microbiota, can lead to the emergence and rapid spread of multidrug-resistant (MDR) bacteria, also referred to as “superbugs” (Davies and Davies, 2010), leading to potential future pathogenic outbreaks. In fact, antimicrobial resistance is projected to cause approximately 10 million deaths, annually by year 2050 (O’Neill, 2014), outpacing cancer, which is the current leading cause of human mortality. It is also pertinent to mention here that secondary infections by nosocomial, or hospital acquired pathogens is on the rise; at least 50% of the deaths reported from the ongoing COVID-19 outbreak were caused from secondary infections (Antibiotic resistance: the hidden threat lurking behind COVID-19^1^^,^^2^). Specifically, respiratory disease caused initially by the COVID-19 viral agent has been shown to rapidly ensue in pneumonia, thus predisposing patients to secondary bacterial infections, and exacerbating underlying cardiac and pulmonary conditions leading to death. Therefore, it is critical to obtain a better understanding on the state of antimicrobial resistance prevalent in environmental representatives of bacterial pathogens, because the environment is a well-known reservoir of pathogens. A central question in our ongoing work is focused on assessing those genomic features that underpin antimicrobial resistances in opportunistic bacterial pathogens in the environment, so that appropriate mitigation strategies to combat these pathogens can be obtained.

Among the bacteria genera that are listed above and relevant to the SRS ecosystem, one genus that deserves further study because of its double life strategy is *Serratia* species, from the Enterobacteria family. Notably, recent genomics analysis has resulted in the division of *Serratia* spp. into the following broad strain categories based on their functional activities: pathogenic, environmental and symbiotic strains (Sandner-Miranda et al., 2018). Furthermore, the environmental strains are divided into three sub-groups: environmental strains that are symbiotic and associated with soil and plants, environmental strains that have been isolated from water and environmental strains that were isolated from food sources. Overall, it was demonstrated that pathogenic and environmental *Serratia* isolates presented a high number of antibiotic resistant genes (ARGs); especially for efflux systems (Levy, 2002; Nelson et al., 2006; Armalytė et al., 2019; Ben Maamar et al., 2020). The nosocomial pathogenic *Serratia* strains possessed a higher degree of acquired resistance genes relative to environmental isolates. However, stress posed by long-term heavy metal contamination is known to exacerbate recruitment and proliferation of ARGs in the native soil microbiota (Cycoń et al., 2019; Kraemer et al., 2019), leading to the main question for this study: are environmental *Serratia* species developing broad antimicrobial resistance activities? To address this overarching question, we performed this case study on environmental representatives of *Serratia* spp., specifically on a newly isolated representative strain (Gendy et al., 2020b), compared to a plethora of other *Serratia* spp., having originated from both environmental and clinical settings.

Note that the environmental *Serratia* species perform a variety of ecosystem services in the environment, including bioremediation of pentachlorophenol (Singh et al., 2009), dichlorodiphenyltrichloroethane (Bidlan and Manonmani, 2002), diesel (Rajasekar et al., 2007), chlorpyrifos (Cycoń et al., 2013), as well as heavy metals to include, nickel (Kannan and Ramteke, 2002), chromium (Mondaca et al., 2002; Campos et al., 2013), molybdate (Yunus et al., 2009; Shukor et al., 2010), lead, cadmium (Cristani et al., 2012), manganese (II) and Hg (François et al., 2012). Some *Serratia* spp., are also well-known plant growth promoting bacteria (PGPB), exhibiting positive impacts by increasing nutrition to the plants, reducing stress and enhancing overall plant productivity (Singh and Jha, 2016). As stated above, despite these attractive environmental and agricultural traits of *Serratia* spp., they are also known to possess pathogenicity, accounting for nosocomial infections of the respiratory tract, urinary tract, surgical wounds and even meningitis (Khanna et al., 2013; Abreo and Altier, 2019; Cristina et al., 2019), specifically caused by *Serratia marcescens*. Therefore, this potential dual-life strategy of *Serratia marcescens* needs to be carefully assessed, specifically for the presence and activities of ARGs, in context to their environmental applications, such as for U and Hg bioremediation.

With this backdrop, we pursued this study keeping in mind the following objectives which are summarized as follows: (1) obtain an environmental *Serratia* isolate resistant to Hg and U; (2) evaluate metabolic potential of the isolated strain and screen how metabolic activity is affected by stress posed by Hg; (3) evaluate the presence and functional activity of antimicrobial resistance using phenomics; and finally, (4) survey the genomic potential of the isolated strain in context to both, metal resistant genes (MRGs) and antibiotic resistant genes (ARGs). Overall, this study provides a broader and comprehensive understanding on the myriad of evolutionary and adaptive traits possessed by an environmental isolate of *Serratia* species, which can be opportunistic pathogens, with special emphasis on carriage of heavy metal and antibiotic resistances. Acquired resistance to multiple antibiotics has been recognized as a global crisis, which needs urgent attention so that a major outbreak, similar to the ongoing global crisis brought about by COVID-19 pandemic, can be avoided. Studies such as this, suggests immediate remedial measures be adopted to obtain mitigation strategies against the development of multidrug resistant bacteria, such as the environmental *Serratia* species, and promulgate strategies in the event a bacterial pathogen outbreak occurs and causes large-scale human fatalities, akin to the COVID-19, which is ongoing, as of this writing.

## Experimental Section

### Isolation and Identification of Strain SRS-8-S-2018

As part of an ongoing study, soil samples were obtained from the SRS Mills Branch (area 101) which is found co-contaminated with total Hg concentrations in the range of 300–700 ng/g dry weight (Xu et al., 2019) as well as U. To isolate heavy metal resistant bacteria, soils were serially diluted and plated onto Lysogeny Broth agar (LBA) containing 5 μg/ml of mercuric chloride; the resulting colonies were picked and further isolated based on their distinct morphologies and pigmentation (Chauhan et al., 2018; Agarwal et al., 2019, 2020a). Plates were incubated aerobically at 30°C in the incubator, and resulting colonies were further isolated, yielding a robust Hg resistant (HgR) strain, labeled as SRS-8-S-2018. Strain identification was performed using 16S rRNA sequencing, as recently reported (Gendy et al., 2020b).

### Characterization of Strain SRS-8-S-2018 for Hg and U Resistance

Hg resistance of strain SRS-8-S-2018 was evaluated by dilution of overnight grown strain to OD600 of 0.2. This inoculum (30 μl) was then added to 270 μl of LB amended with incremental concentrations (0–30 μg/ml) of mercuric chloride. OD_600_ was then evaluated every 3 h for up to 48 h at 30°C using the Bioscreen C microbial growth analysis system (Growth Curves USA, Piscataway, NJ, United States), as shown previously (Agarwal et al., 2019). This minimum inhibitory concentration (MIC) assay was performed three times, and the average values are reported. Controls were also run on plates that did not contain Hg and compared under the same growth conditions.

To evaluate resistance against uranium, our recently developed plate MIC method was used because U is known to cause precipitation in liquid media causing turbidity which interferes with monitoring of bacterial growth (Agarwal et al., 2020b). Briefly, strain SRS-8-S-2018 was prepared taking a single colony inoculated into LB broth overnight and then diluted to an OD_600_ of 0.1. Further, a 10-fold dilution series was prepared up to 10^–5^ dilution range and plated onto LB plates containing U in concentrations of 2, 4, 5, and 6 mM, using Uranyl nitrate hexahydrate. Plates were incubated at 30°C and photographs were taken after 48 hours of growth to identify MIC against U.

### Physiological Response of Strain SRS-8-S-2018 in the Presence of Hg

Inoculum was prepared in LB broth with a single colony of strain SRS-8-S-2018, incubated at 30°C until OD_600_ reached 0.4. Then the cells were washed five times with 0.9% NaCl to eliminate any nutrients originating from previous growth in LB broth. This cell suspension (120 μl) was used as inoculum for establishing Biolog^®^ EcoPlates^™^, as suggested by the manufacturer (Biolog Inc., Hayward, CA, United States), such that, each well contained a cell concentration of 10^–5^, which was checked by microscopic evaluation of cell density. The Biolog^®^ EcoPlates^™^ consists of 96-well microtiter plates with three replicates of 31 common substrates utilized by soil microbiota, thus are ecologically relevant for microbial physiological and metabolic activity assessment (Preston-Mafham et al., 2002). A separate set of EcoPlates was established, containing Hg at 3 different concentrations to assess the strain’s physiological response; these concentrations were 1, 3, and 5 μg/ml mercuric chloride, respectively. The resulting absorbance was measured via plate reader at 590 nm (spectrophotometer-SpectraMax-M5), every 24 h over a 20-day period. Obtained data was then plotted using Microsoft Excel and further analyzed. Average well color development obtained from the Biolog^®^ EcoPlates^™^ was estimated as shown before (Gamo and Shoji, 1999; Deng et al., 2011; Gryta et al., 2014).

### Phenotype Microarray (PM) Analysis on Strain SRS-8-S-2018

Phenotype MicroPlate^™^ (PM) from Biolog is an array of 96 well microtiter plate, where each set of 4 wells contain the same antibiotic in increasing increments along with the needed minimal medium components and specific dye. The arrays can provide resistance patterns against 41 antibiotics belonging to 10 different chemical classes, e.g., aminoglycosides, β-lactams, lincosamides, synthetic antibiotics, glycopeptides, tetracyclines, amphenicols, macrolides, sulfonamides, and rifamycins. The antibiotic resistance of bacteria was assessed using Biolog PM11C and PM12B MicroPlate^™^ plate assays, as suggested by the manufacturer; layout of antibiotics is as detailed by the manufacturer for the Biolog^®^ EcoPlates^™^ (Biolog Inc., Hayward, CA, United States)^3^. To initiate the PM assay, strain SRS-8-S-2018 was grown overnight at 30°C on an LB agar medium. A single colony was then picked and inoculated into 20 ml of the inoculating fluid [IF-10a GN Base inoculating fluid (1.2×), Biolog Inc.]. Resulting cell density was measured by a plate reader according to the Biolog protocol (Biolog Inc., Hayward, CA, United States), followed by inoculation of the cell suspension into plates (100 μl/well) and incubated at 30°C for 36 hours. OD_590_ was collected every 15 min to determine color shifts resulting from microbial growth in the wells reflected of phenotypic differences and sensitivity/resistance to different antibiotics. Collected data was organized and plotted in Microsoft Excel.

### Statistical Analysis of BIOLOG Data

Data obtained from Biolog^®^ EcoPlates^™^ over the 7-day period was analyzed using PRIMER v6 (PRIMER-E Ltd.). Statistical relationships were evaluated by running non-metric multi-dimensional scaling (NMDS) analysis, where the different concentrations of substrates utilized in the presence and absence of Hg amendments were log transformed [(Log (*X* + 1)], Hg treatments were used as variables, as appropriate and scaled by maximum data point.

### Nucleotide Sequence Accession Number

The Whole Genome Shotgun project of *Serratia* sp. SRS-8-S-2018 reported in this study has been deposited at DDBJ/ENA/GenBank under the Accession VHNF00000000^4^; BioProject: PRJNA550097^5^; BioSample: SAMN12107717^6^. The version described in this research is version VHNF00000000.1.

### Genomic Analysis of Strain *Serratia* sp. SRS-8-S-2018

To understand the genomic characteristics of strain SRS-8-S-2018 in reference to MRGs and ARGs, a single colony of the bacterium growing on LB plate, amended with Hg, was inoculated into liquid LB media and incubated overnight at 30°C in a shaker at 100 rpm. After overnight growth, the media was centrifuged at 7,500 rpm for 5 min to obtain a pellet, which was then used for DNA extraction with ZR fungal/bacterial DNA kit (Zymo Research, Irvine, CA, United States) and sequenced using an Illumina HiSeq2000 instrument. Assembly and annotation on the obtained contigs were performed, as reported in our recent study (Gendy et al., 2020b). Default settings were used in all the genomic analysis, unless otherwise stated.

Taxonomic affiliation was conducted using the Onecodex workflow (Minot et al., 2015), which is based on the identification of typically 17–31 bp short sequences, that are found unique to a specific taxa within the inputted sequence reads. Based on the collection of *k*-mers found in a given read, the whole genome sequence is then assigned to a taxon. Phylogenomic analysis was run using the default value of *k* = 31 for the taxonomic assessment of strain SRS-8-S-2018 using the Onecodex database. To obtain a physical map of the genome, CGView Comparison Tool was used with default settings (Grant et al., 2012). The genome, with a 106× coverage was annotated and the genes were predicted using Rapid Annotations using Subsystems Technology-RAST (Aziz et al., 2008), Prokaryotic Genomes Automatic Annotation Pipeline (PGAAP), version 2.0 (Tatusova et al., 2016), the Pathosystems Resource Integration Center (PATRIC, version 3.6.5) (Wattam et al., 2017) or Integrated Microbial Genomes (IMG) system (Chen et al., 2019). Average nucleotide identity (ANI), and average amino acid identity (AAI) was also obtained via EDGAR (Blom et al., 2009) and EzBioCloud pipelines (Yoon et al., 2017). ANI is a measure of the genomic resemblance of two different bacterial species and typically, ANI values between genomes of the same species are 95% or beyond (Goris et al., 2007).

The *merR* gene taxonomy in strain SRS-8-S-2018 was evaluated using comparative functions embedded within the PATRIC pipeline. Venn diagram and phylogenomic comparisons were obtained using the EDGAR pipeline, version 2.0 (Blom et al., 2009). After EDGAR analysis, the Newick phylogenomic tree file was downloaded and an amino acid based tree was constructed using MEGAX (Wattam et al., 2017; Kumar et al., 2018). Island Viewer (Bertelli et al., 2017) was used to identify genomic islands (GEIs) (Yung et al., 2015), within the genome of SRS-8-S-2018. Remnants of phage DNA, also called prophage, present in the whole genome sequence of SRS-8-S-2018 were located using the prophage hunter (Song et al., 2019).

## Results and Discussion

### Isolation, Identification and Characterization of Metal Resistance Potential of Strain SRS-8-S-2018

**FIGURE 1:**
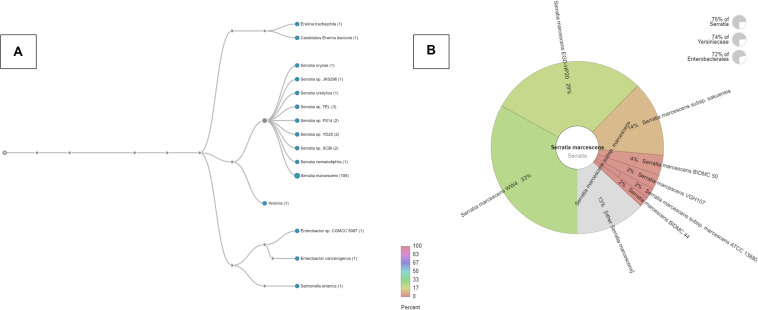
Phylogenomic assessment of *Serratia* sp. strain SRS-8-S-2018 providing evidence for close affiliation with *Serratia marcescens*. Shown are **(A)** phylogenomic analysis of strain SRS-8-S-2018 using the default settings in the Onecodex workflow; **(B)** strain-level taxonomic affiliation with other *S*. *marcescens* that revealed 33% sequence reads belonged to *S*. *marcescens* strain WW4, followed by 29% of *S*. *marcescens* strain EGD-HP20.

Our recent studies suggest that the metalliferous SRS soils may be serving as a reservoir for the recruitment and proliferation of metal and antimicrobial resistances in the native microbial communities (Agarwal et al., 2019, 2020a, 2020b; Gendy et al., 2020a, b; Pathak et al., 2020), thus presenting risks to the ecological processes and public health. Overall, this study further builds upon evidence to show that the SRS contaminated soils inherently harbor higher antibiotic resistance, relative to the reference soils, and thus driving antimicrobial resistance to the native microbiota, including several known pathogens (e.g., *Burkholderia*, *Ralstonia*, *Massilia*, *Acinetobacter*, and *Pseudomonas* (Stepanauskas et al., 2006; Wright et al., 2006; Thomas et al., 2020). To assess if carriage of metal resistant genes (MRGs), and antibiotic resistant genes (ARGS) is a rampantly widespread trait, especially within opportunistic bacteria native to the SRS soils, this study was conducted on soils collected from a different site (101), relative to that reported by our group recently (Pathak et al., 2020). Specifically, soils from a highly contaminated site- SRS 101 were serially diluted and spread plated onto LB agar plates supplemented with 5 μg/ml mercuric chloride. Site 101 has been shown to be co-contaminated with a variety of different heavy metals, such as Hg and U (Edwards et al., 2014). Plates were incubated at 30°C, for 48 hours and colonies that appeared on the plates were further purified resulting in a vigorous Hg-resistant (HgR) strain, labeled as SRS-8-S-2018. Identification of the isolated strain using 16S rRNA gene sequencing revealed closest taxonomic affiliation with *S. marcescens* (Gendy et al., 2020b). Specifically, phylogenomic analysis, as stated later in this study, revealed 71.62% of obtained genome reads affiliated with *S. marcescens* (Figure 1A); at the species level, 33% of the reads belonged to *S*. *marcescens* strain WW4, followed by 29% of *S*. *marcescens* strain EGD-HP20 (Figure 1B).

**FIGURE 2:**
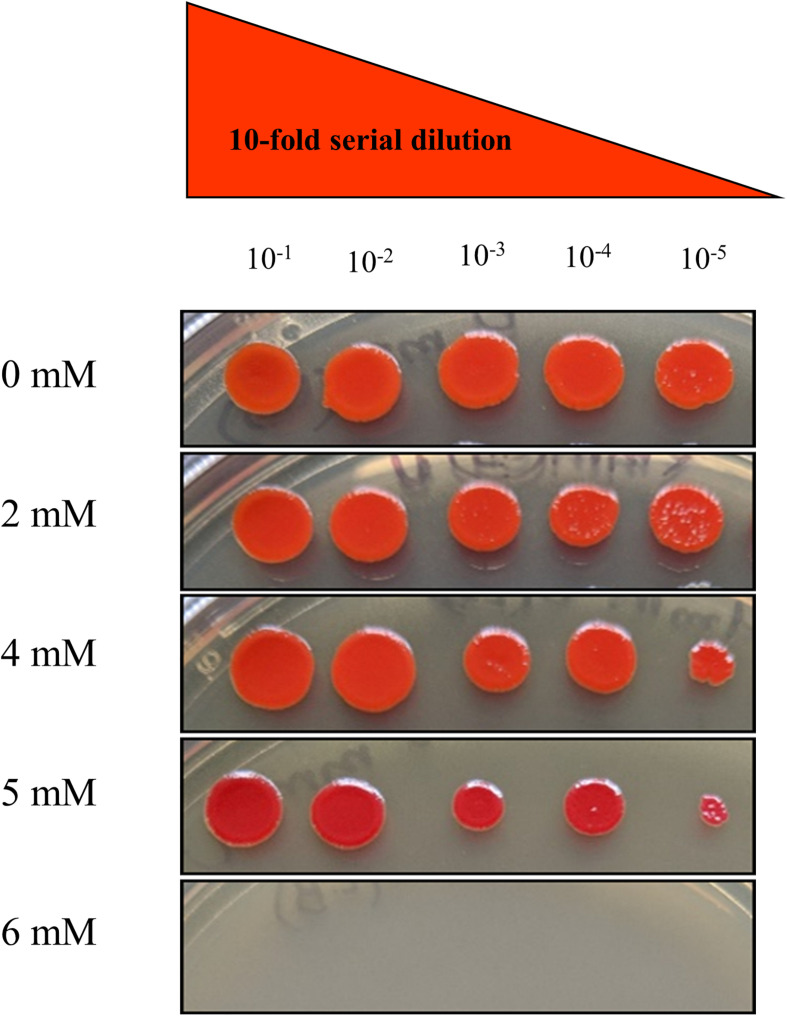
Minimum inhibitory concentration of *Serratia* sp. strain SRS-8-S-2018 on different concentrations of uranium (U) obtained from the plate MIC method, revealing resistance of U over 1–5 mM.

To determine the resistance potential of strain SRS-8-S-2018 against Hg, the bacteria was grown in LB broth, supplemented with different concentrations of Hg; these results were presented in our recent study (Gendy et al., 2020b). It was observed that strain SRS-8-S-2018 could resist up to 10 μg/ml Hg, which revealed a strong Hg resistance (HgR) phenotype of strain SRS-8-S-2018. To test for resistance against uranium (U), a plate MIC method was used, which showed resistance of strain SRS-8-S-2018 at 2, 4, and 5 mM but strain was sensitive at 6 mM U, respectively (Figure 2). Note that *S. marcescens* has previously been shown to be resistant to U under both aerobic and anaerobic environments (Kumar et al., 2011; Choudhary et al., 2012; Nongkhlaw et al., 2012; Newsome et al., 2015). To further understand the Hg and U-resistant ability of this strain, physiological and genomic studies were then performed.

### Physiological Response of *Serratia marcescens* Strain SRS-8-S-2018 in the Absence and Presence of Hg Amendments

**FIGURE 3:**
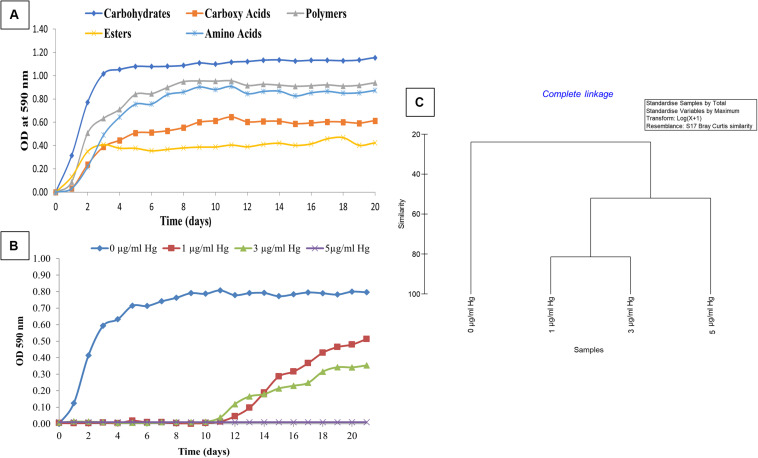
Physiological assessment of *Serratia* sp. strain SRS-8-S-2018 using the Biolog^®^ EcoPlates^™^ system. Shown are **(A)** physiological activity of strain SRS-8-S-2018 measured as average well color development (AWCD) against 5 different classes of growth substrates; **(B)** average well color development (AWCD) of strain SRS-8-S-2018 in the presence of 1, 3, and 5 μg/ml of Hg amendments; **(C)** cluster analysis of non-metric multi-dimensional scaling (NMDS) based on Bray–Curtis dissimilarities between different Hg amendments on strain SRS-8-S-2018.

Biolog^®^ EcoPlates^™^, containing 31 distinct nutrient sources, was used to evaluate the metabolic and physiological response of strain SRS-8-S-2018 in the absence and presence of Hg amendments, as a representative heavy metal contaminant. The cellular response in this assay was monitored as a function of color change of the tetrazolium salt when substrates in each well are utilized by cellular respiration, resulting in the production of a purple formazan dye. Higher the substrate utilization, the more intense the color of the formazan produced and by inference, metabolic activity of the cell. Kinetic growth response curves were obtained for each growth substrate and plotted to assess cellular phenotype comparisons by calculation of the average well color development (AWCD), which revealed that *Serratia* sp. SRS-8-S-2018 utilized different sources of carbon in the following order carbohydrates > polymers > amino acids > carboxy acids > esters (Figure 3A), respectively. Esters initially were preferred over carboxy acids and amino acids but after 3 days, the above stated trend held up until the experiment ended at 20 days. Specific to different carbon sources, it was found that strain SRS-8-S-2018 preferred the following carbon substrates: Specific to different carbon sources, it was found that strain SRS-8-S-2018 preferred the following carbon substrates: D-Mannitol, i-Erythritol, γ-Amino Butyric Acid, L-Asparagine, *N*-Acetyl-D-Glucosamine, and Tween 40 (data not shown).

To understand how Hg stress may impact metabolic potential, growth of strain SRS-8-S-2018 was evaluated in the presence of increasing concentrations of Hg, which showed that at both, 1 and 3 μg/ml Hg concentrations, the strain’s metabolic potential (AWCD) became inhibited; the strain started to use substrates after a lag of almost 12 days (Figure 3B). However, at an Hg concentration of 5 μg/ml, the strain was unable to utilize any of the carbon substrates, despite the strain’s potential to resist up to 10 μg/ml Hg based on MIC values (Gendy et al., 2020b). This difference in the MIC and physiological activity response in the presence of Hg amendments can be explained on the basis that the higher MIC in LB broth, which is a richer nutrient source relative to those present in the Biolog^®^ EcoPlates^™^. Regardless, this extensive physiological characterization demonstrated that *Serratia* sp. SRS-8-S-2018 used an average of 55.84% of the 31 different nutrient sources tested without Hg stress (Table 1); conversely, in the presence of 1 μg/ml Hg, this response became inhibited at 45.16% of total substrates; at 3 μg/ml Hg even fewer substrates were utilized- at an average of 32.25%, while at a concentration of 5 μg/ml Hg, total physiological activity of the Hg-resistant strain ceased (Figure 3B and Table 1), despite the robust Hg resistance ability of SRS-8-S-2018. Interestingly, the utilization rates of amino acids, such as L-Asparagine, L-Alanine, and L-Serine, relative to other substrates significantly decreased upon exposure across all treatments (data not shown), most likely due to binding of Hg to amino acids which reduces their availability and hence physiological activity. It could also be that metabolic pathways for the utilization of these amino acids are hampered by Hg toxicity. It has been well-established that mercury, the most toxic heavy metal toxicity, is mainly owed to its high degree of affinity to the sulfhydryl ligands in amino acids, binding to which causes alteration in protein structure along with loss of protein function. Overall, this part of the study suggests that Hg can interfere with the bacterial physiological response, even on an organism that is resistant to Hg. Therefore, continued addition of Hg can have detrimental impacts to microbially mediated ecosystem services, such as biogeochemical cycling of nutrients. This is a significant finding from an environmental standpoint, which has not been demonstrated before, to our knowledge.

**TABLE 1:** Individual substrate utilization patterns shown by *Serratia* sp. strain SRS-8-S-2018 in the absence and presence of different Hg concentrations using Biolog^®^ EcoPlates^™^.

Individual substrate	0 μg/ml Hg	1 μg/ml Hg	3 μg/ml Hg	5 μg/ml Hg
Pyruvic acid methyl ester	+	+	+	−
Tween 40	+	+	+	−
Tween 80	+	+	+	−
α-cyclodextrin	−	−	−	−
Glycogen	−	−	−	−
D-cellobiose	−	−	−	−
α-D-lactose	−	−	−	−
β-methyl-D-glucoside	+	+	+	−
D-xylose	−	−	+	−
*i*-erythritol	+	+	+	−
D-mannitol	+	+	+	−
*N*-acetyl-D-glucosamine	+	+	+	−
D-glucosaminic acid	−	−	−	−
Glucose-1-phosphate	+	+	−	−
D,L-glycerol phosphate	−	−	−	−
D-galactonic acid lactone	−	−	−	−
D-galacturonic acid	+	+	+	−
2-hydroxybenzoic acid	−	−	−	−
4-hydroxy benzoic acid	−	−	−	−
γ-amino butyric acid	+	+	+	−
Itaconic acid	−	−	−	−
α -keto butyric acid	−	−	−	−
D-malic acid	+	+	−	−
L-arginine	−	+	−	−
L-asparagine	+	+	−	−
L-phenylalanine	+	−	−	−
L-serine	+	+	−	−
L-threonine	−	−	−	−
Glycyl-L-glutamic acid	+	−	−	−
Phenylethylamine	+	−	−	−
Putrescine	+	−	−	−

*A positive sign (+) indicates strain utilization of carbon source and the negative sign (−) indicates inability of the strain to utilize the substrate source.*

Statistical analysis of the strain’s physiological response to Hg amendment was assessed using Primer v6, which is shown in Figure 3C. Cluster analysis clearly showed that in the absence of Hg, the strain’s metabolic activity was statistically different relative to Hg treatments. Specifically, the metabolic activity profiles at 1 μg/ml Hg and 3 μg/ml Hg clustered together and away from the highest tested concentration of 5 μg/ml Hg, mirroring the trends shown in Figures 3A,B. This confirms the observation that Hg can interfere with the physiological and metabolic response, even in an organism that harbors Hg resistance. Therefore, continued addition of Hg will likely have significant detrimental impacts to microbially mediated nutrient cycling processes; even to those microorganisms that have developed tolerance/resistance against heavy metals, such as Hg. Arguably, microbiota that are unsuccessful in the recruitment of resistant gene determinants against heavy metals will have likely been removed from a contaminated soil habitat and ecosystem services rendered by the outcompeted bacteria are either lost, diminished or taken over by the surviving metal resistant bacteria. To further understand the gene determinants for heavy metal resistance, genomic studies were performed on strain SRS-8-S-2018.

### Genome-Centric Assessment of *Serratia marcescens* Strain SRS-8-S-2018

**FIGURE 4:**
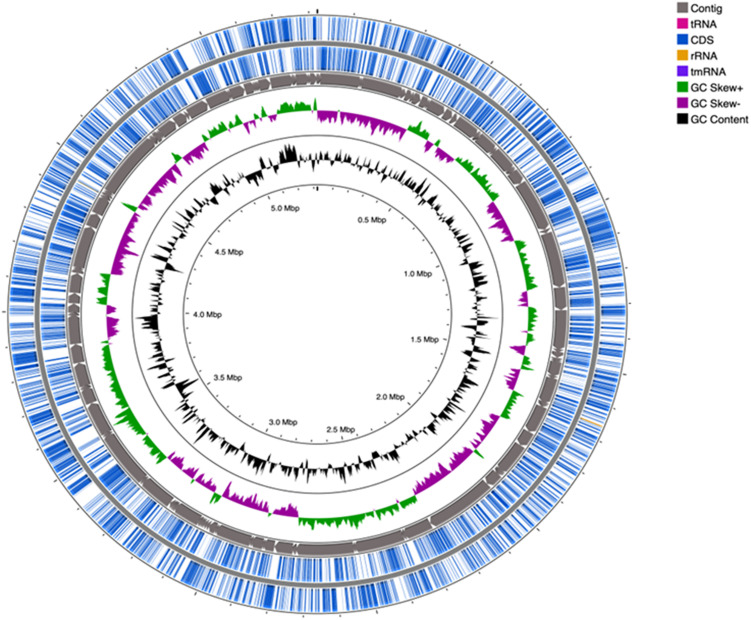
Genomic mapping of *Serratia* sp. strain SRS-8-S-2018. Shown is a circular genome map of strain SRS-8-S-2018, obtained using the default settings within the CGView Comparison Tool. The map consists of 5 circles from inside out; first GC content, the second contain GC positive and negative skew, the third contains contigs, the fourth and fifth contain coding sequences (CDS) and tRNA, and rRNA, respectively.

Genomic sequences of strain SRS-8-S-2018, with a coverage of 106× were obtained (Gendy et al., 2020b), and characterized by the following parameters: contig count (159); total length (5,323,630 bp); N50 (101301 bases); L50 (17); an average GC content of 59.48%; a coding sequence of 5,261 proteins (CDS) and 55 genes for transfer RNA (tRNA) and 3 genes for rRNA, respectively. Figure 4 shows a circular genomic map of strain SRS-8-S-2018 constructed with CGView Comparison Tool. Gene prediction performed using PATRIC, RAST and NCBI pipelines revealed that strain SRS-8-S-2018 harbored a total of 5095 protein coding genes; 80.29% of these genes were annotated as protein coding genes with predicted functions; 27.68% genes were annotated as protein coding genes with enzyme production and 31.14% genes were associated with KEGG pathways, respectively. Moreover, 82.17% of the protein coding genes were annotated with COGs, i.e., clusters of orthologous groups of protein; COGs represent an ortholog or direct evolutionary counterpart among bacterial genomes as they evolve over time.

As stated before, phylogenomic analysis of strain SRS-8-S-2018 relative to other sequenced *Serratia* species revealed closest taxonomic affiliation with *Serratia* species and other genera, such as *Salmonella* (Figure 1A). Specifically, onecodex analysis resulted in the assignment of 33% contigs from strain SRS-8-S-2018 to *S. marcescens* WW4, followed by 29% to *S. marcescens* EGD-HP20 and 14% with *S. marcescens* subsp. *Sakuensis*, respectively (Figure 1B); these are all environmental strains not directly related to their hospital or nosocomial counterparts. Similar result was demonstrated by EDGAR analysis (data not shown), in which a phylogenomic tree of strain SRS-8-S-2018 was built with 15 *Serratia* genome sequences, build out of a core of 1195 genes per genome, 17925 in total. The core had 321435 amino acid residues/bp per genome, 4821525 in total. Furthermore, average nucleotide identity (ANI) analysis mirrored the previous results such that *S. marcescens* sp. WW4 was the closest strain to *Serratia* sp. SRS-8-S-2018 with a 99.7% value followed by *S. marcescens* sp. EGD HP20 (99.68%) and *Serratia* sp. FS14 (99.45%), respectively (Supplementary Figure S1). Note that the ANI calculator estimates the average nucleotide identity using both best hits (one-way ANI) and reciprocal best hits (two-way ANI) between two genomic datasets, as calculated by Goris et al. (2007). Typically, the ANI values between genomes of the same species are above 95% and therefore, strain SRS-8-S-2018 is assigned to *S. marcescens*.

After the taxonomic assessment of strain SRS-8-S-2018, further functional characterization was performed using RAST, which revealed the presence of 585 subsystems with the following top six categories, genes in parenthesis: amino acid metabolism (428); carbohydrate metabolism (391); vitamin and cofactor metabolism (197); protein metabolism (116); membrane transport (136) and fatty acids and lipids (136), respectively. Similar results were shown by PATRIC based annotation also revealed the presence of genes (in parenthesis) for metabolism (967); energy production (310); protein processing (255); stress response/defense/virulence (207); membrane transport (174); cellular processes (157); DNA processing (109); 92 genes were also found for resistance to antibiotics and toxic compounds. Also identified are 31 genes shown homologous with prophages, transposable elements and plasmids, indicating the receptivity or interactions of strain SRS-8-S-2018 with mobile genetic elements.

Of particular relevance are several gene homologs that have previously been shown to be involved in the resistance against heavy metals/radionuclides (genes in parenthesis), including efflux systems (48) and membrane transporters (121), which likely facilitates survival of strain SRS-8-S-2018 in a metalliferous soil habitat. These findings are similar to our previous genomic analysis of another *Serratia* species- strain S1B, isolated from the Savannah River Swamp System soils (Pathak et al., 2018). Note that the SRSS site is located at the confluence of Four Mile Creek and the Savannah River, which received liquid Hg (Hg) effluents from a chloralkali facility near Augusta, GA, United States, until the 1980s. Strain S1B, native to these Hg-contaminated soils, was found to harbor a genomic size of approximately 7,710,841 bases^7^ with 7,075 coding sequences and a GC content of 45.9%. A total of 585 subsystems were annotated from strain S1B with genes (number in parentheses) for membrane transport (282), stress response (221), metabolism of aromatic compounds (173), motility and chemotaxis (9), among gene homologs shown to be involved in the resistance against heavy metals, similar to results presented here for strain SRS-8-S-2018. Collectively, these analyses of *Serratia* species strain SRS-8-S-2018 revealed the presence of several genome-enabled metabolic and catabolic processes, which likely play a significant role in the colonization and strain’s survival within the metalliferous SRS soil habitat; similar to previous studies that we have conducted on long-term contaminated sites (Olaniran et al., 2013; Ojuederie and Babalola, 2017; Chauhan et al., 2018).

**FIGURE 5:**
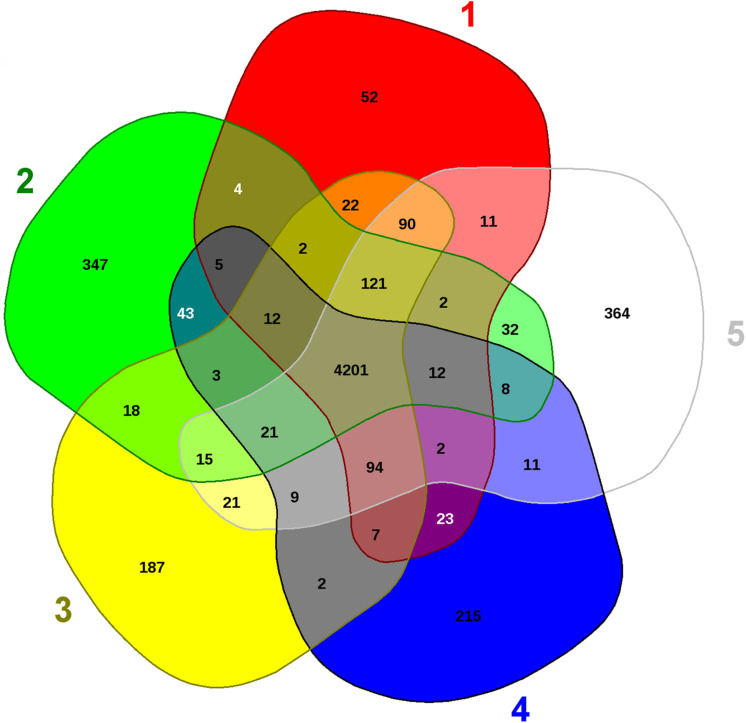
Comparative genomic assessment of *Serratia* sp. strain SRS-8-S-2018. Shown are the whole genome sequence-based comparative analysis of strain SRS-8-S-2018 with four closest taxonomic relatives obtained using default settings in the EDGAR pipeline. Venn diagram sectors belong to **(1)** *S. marcescens* EGD_107; **(2)** *S. marcescens* VGH107; **(3)** *S. marcescens* WW4; **(4)** *S*. species FS14, and **(5)** Strain SRS-8-S-2018. The number of singleton genes appear in red, green, yellow, blue, and white areas for strains 1–5 listed above -along with their core genomes (centered gray area).

Further analysis was performed using comparative genomics to evaluate evolutionary and adaptive traits of strain SRS-8-S-2018 with the closest taxonomic strains- *Serratia* species WW4, EGD_HP20, VGH107, and FS14, as shown by EDGAR analysis (data not shown). This revealed the presence of 364 distinct genes present only in strain SRS-8-S-2018 relative to the other closely associated *Serratia* strains (Figure 5). Similarly, distinctive genes were also identified in the other four strains analyzed, shown in parenthesis: *Serratia marcescens* EGD_HP20 (52); *S. marcescens* VGH107 (347); *S*. *marcescens* WW4 (187), and *S*. species FS14 (215), respectively. Even though 364 genes present in SRS-8-S-2018 (#5 sector in Figure 5), made up only about 7% of the total genome size of the strain, but this set of genes are representative of “distinct” determinants for efflux, metal resistance, universal stress proteins, cytochromes, drug resistance, transport proteins as well as transposases. Moreover, many of the unique genes in SRS-8-S-2018 were classified as hypothetical proteins, functions of which are unknown at this time.

It is also noteworthy that the strain harbored several genes that have been implicated in diverse roles pertaining to plant promoting activities, such as: phosphate metabolism (*phoU*, *pstB*, *pstA*, *pstC*, and *pstS*); spermidine synthase and N1-acetyltransferase genes (data not shown). These traits are similar to other *Serratia* species (Matteoli et al., 2018), and may render a plant growth promoting role of strain SRS-8-S-2018 in its native soil habitat, simultaneously reducing the metal toxicity. Overall, this comparative genomic analysis confirmed a strong genome-enabled bioremediative as well as plant growth promoting potential of strain SRS-8-S-2018.

### Genomic Islands (GEIs) in *Serratia marcescens* Strain SRS-8-S-2018

**FIGURE 6:**
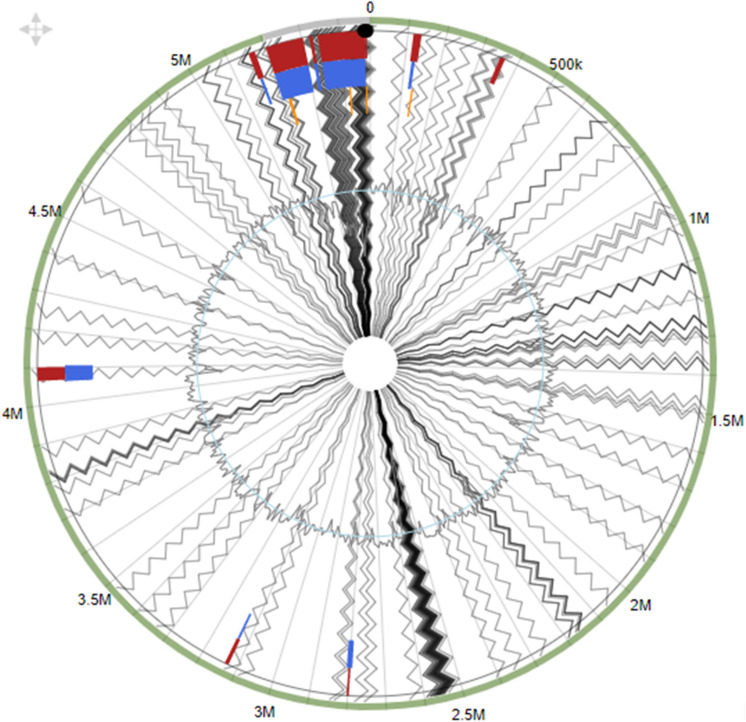
Genomic islands identified within the whole genome sequence of *Serratia* sp. strain SRS-8-S-2018. Shown are putative genomic islands (GEIs) harbored by strain SRS-8-S-2018 relative to the genome of *S. marcescens* WW4, used as the reference strain. The outer black circle represents the scale line in Mbps and GEIs obtained from each of the following methods are shown in color: SIGI-HMM (orange), IslandPath-DIMOB (blue), and integrated detection (red), respectively.

Using the integrated prediction method to identify GEIs, strain SRS-8-S-2018 was shown to harbor 13 different GEIs, ranging in size from 125,063 to 5,237 bp (Figure 6), when *S. marcescens* WW4 was used as the reference genome. Note that genomic islands is a universal trait in many environmental isolates we have studied to date (Chauhan et al., 2018; Pathak et al., 2020); GEIs are beneficial gene segments recruited by host bacteria from their external environment via horizontal gene transfer (HGT) mechanisms (Pérez-Pantoja et al., 2013). Typically, GEIs have been shown to be associated with host-beneficial adaptive traits such as bioremediation, virulence, antibiotic resistance and metabolism. Overall, these GEI-encoded traits can be binned under the following four major categories (Bertelli et al., 2019): (1) metabolic islands (MIs), which are a set of genes for secondary metabolite biosynthesis; symbiotic islands (SIs), which are those set of genes that facilitate symbiotic associations with other micro and/or macroorganisms; pathogenicity islands (PAIs), coding for virulence or disease causing genes; and resistance islands (RIs), rendering resistance for antibiotics and other bacteriostatic/bactericidal agents.

Overall, this analysis led to the identification of several genomic islands in strain SRS-8-S-2018, with gene functions previously identified for metal resistance, efflux and transposons. This strongly indicates a likelihood that GEIs are probably recruited via HGT to promote survival in a heavy metal contaminated soil habitat (Bertelli et al., 2017). These findings are similar to our previous reports from *Serratia* strain B1, which also revealed a repertoire of biodegradative genes, several occurring on genomic islands (Pathak et al., 2018). The presence of these GEIs in SRS-8-S-2018 also provides clues into the strain’s genome plasticity, introduced by mobile genetic elements including integrases or transposases, which were also identified.

Notably, one mechanism for GEIs to be incorporated into bacterial genomes is through bacteriophage attack, which can leave phage remnant DNA, or prophage, integrated into the bacterial genome. Thus, bacteriophage attacks is a major mechanism that influences bacterial evolution, resulting in the recruitment of genomic traits beneficial to the host species, such as virulence factors, antibiotic resistance mechanisms and bioremediative functions (Bernheim and Sorek, 2018). When presence of prophage was evaluated in strain SRS-8-S-2018, 156 candidate genes were identified in this category, as shown in Supplementary Table S1. Specific details on the top 5 phage-associated genes are as follows (sizes in parenthesis): *Salmonella* phage FSL SP-004 (68,109 bp); *Salmonella* phage 29485 (52,465 bp); *Nocardia* phage NBR1 (47,541 bp); *Edwardsiella* phage PEi26 (47,062 bp) and *Klebsiella* phage 4 LV-2017 (38,728 bp), respectively. In fact, 12 different *Salmonella*-specific prophages were found associated in the genome of strain SRS-8-S-2018 followed by 5 from *Klebsiella* spp. Conversely, only 1 phage X20 was identified in the genome of strain SRS-8-S-2018 that was related to *Serratia* spp., which indicates strain SRS-8-S-2018 was more responsive to *Salmonella*-specific phage interactions relative to its own genus. Because *Salmonella* possess pathogenic traits, it is likely that pathogenicity of *Serratia* spp. are transmitted from *Salmonella* into *Serratia* via HGT mechanisms, but this observation is based only on genomic analysis with no rigorous functional proof available as of this writing. This observation, however, also goes well in line with the taxonomic similarity between the strain’s genome with *Salmonella* spp., albeit at only 0.68% (data not shown). In this context, genome-wide analysis of *Serratia* species have recently established that the environmental *Serratia* representatives have commonalities pertaining to the high number of antibiotic resistant genes relative to the hospital or nosocomial strains (Sandner-Miranda et al., 2018) and it appears that the environmental isolates are also equally potent in their antimicrobial activities.

### Metal Resistant Genes (MRGs) of *Serratia marcescens* Strain SRS-8-S-2018

Genomic mining of *Serratia* sp. SRS-8-S-2018 also revealed several genes that likely enable bacterial adaptability and survival to survive in a contaminated environment. For example, it was found that the strain contained several gene homologs for heavy metal and biocide resistance such as efflux proteins [e.g., resistance, nodulation, and protein-family (RNDs); outer membrane channel TolC], and heavy metal-responding transcription regulators. Furthermore, the SRS-8-S-2018 strain genome had significant numbers of ABC-type transport and heavy metal detoxication proteins, which could maintain homeostasis of metals – all of these adaptations likely contribute for environmental and evolutionary response of strain SRS-8-S-2018 and survival in a metalliferous soil habitat.

One major mechanism underpinning microbial resistance against both heavy metals and antibiotics is by efflux of these compounds outside of the cellular environment, as soon as the cells sense their presence (Levy, 2002; Nies, 2003; Blanco et al., 2016; Buffet-Bataillon et al., 2016). Strain SRS-8-S-2018 possessed at least 48 genes related to different efflux groups such as ABC-type efflux, multidrug efflux, RND efflux system etc. (data not shown). Therefore, efflux mechanisms may be one major response of strain SRS-8-S-2018 to survive in a highly metalliferous soil habitat. Toward this end, Hg-resistance is also likely efflux based in the strain. Genome mining revealed that the strain did not support a complete *mer* operon but only harbored the transcriptional regulator, *Mer*R family (fig| 613.215.peg.349) (Supplementary Figure S2A), along with the heavy metal sensor histidine kinase (HMHK) and the DNA-binding heavy metal response regulator (HMRR). The *merR* gene in strain SRS-8-S-2018 was taxonomically closest to *S. marcescens* WW4 and *S. marcescens* EGD-HP20 (Supplementary Figure S2B); these strains were also identified closest relatives in the phylogenomic based analysis shown in Figures 1A,B. Therefore, one central question arises from the lack of a complete *mer* operon in strain SRS-8-S-2018- what mechanism(s) render Hg resistance to *Serratia marcescens* strain SRS-8-S-2018?

To this end, previous body of information revealed that not all HgR bacteria support a complete *mer* operon. The *mer* operon has origins in geothermal environments (Boyd and Barkay, 2012), and has evolved from being a constitutively expressed system made up of simple genetic elements to a highly regulated and complex operonic system- the complete *mer* operon. We are tempted to speculate that Hg resistance in strain SRS-8-S-2018 is likely mediated via efflux mechanisms, as proposed elsewhere. In fact, studies have found energy-dependent efflux systems to render resistance to many heavy metals, including cadmium, zinc, nickel, cobalt, and copper (Reyes et al., 1999). In a seminal report, several *mer* negative but HgR bacteria exhibited decreased resistance to Hg when amended with the protonophore- carbonyl cyanide m-chlorophenylhydrazone (CCCP) (Reyes et al., 1999). It may also be that strain SRS-8-S-2018 harbors genes which are not homologous to recognized *mer genes* and HgR is a mechanism driven by potentially novel genes; we are currently conducting more work to resolve these possibilities in strain SRS-8-S-2018 as it relates to Hg resistance. Regardless of these possibilities, the presence of abundant MRGs in strain SRS-8-S-2018 provides for a deeper understanding on soil survival strategies.

### Antimicrobial Resistant Genes (ARGs) of *Serratia marcescens* Strain SRS-8-S-2018

**FIGURE 7:**
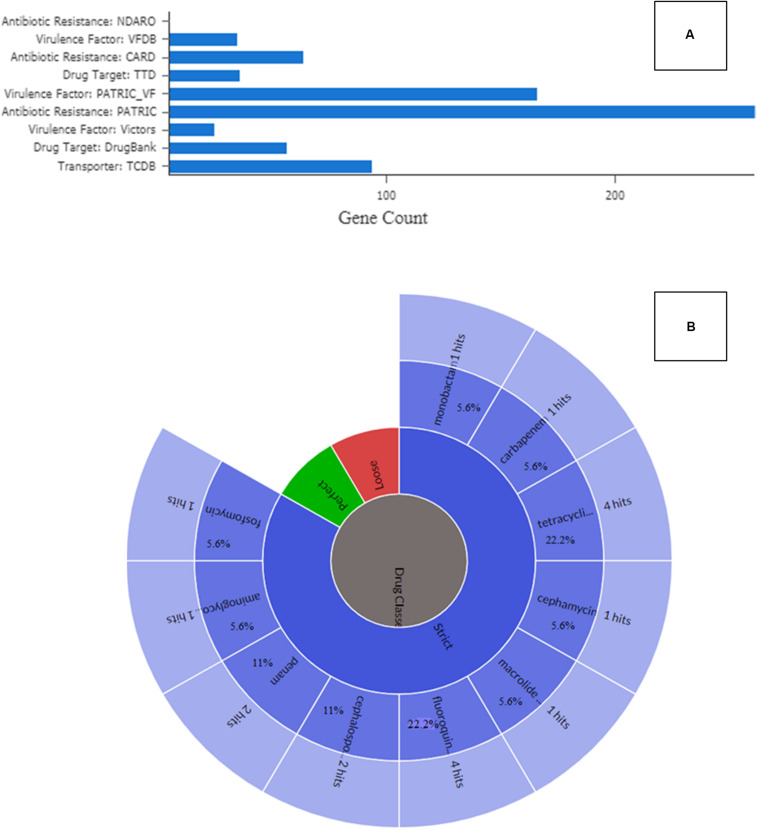
Annotation and gene prediction analysis of *Serratia* sp. strain SRS-8-S-2018. Shown are **(A)** identification of specialty genes identified in *Serratia* sp. strain SRS-8-S-2018 after annotation and gene prediction using the PATRIC workflow; **(B)** presence of antibiotic resistant genes (ARGs) from strain SRS-8-S-2018 using the Comprehensive Antibiotic Resistance Database (CARD).

To validate the phenomic response indicating carriage of broad antibiotic resistance in SRS-8-S-2018, a comprehensive survey of antibiotic resistance genes (ARGs) was performed employing PATRIC, CARD, and RAST pipelines. This bioinformatic analysis revealed a plethora of 104 ARGs in *Serratia* sp. SRS-8-S-2018. Specifically, PATRIC analysis revealed the presence of several genes classified under speciality category, that included 261 genes for transporters, 200 genes for drug targets, 173 genes for virulence factors, and 102 genes for antibiotic resistance, respectively (Figure 7A). This points to the repertoire of speciality genes, many related to virulence and ARGs, that are recruited by this soil-borne isolate as its arsenal againts multiple antimicrobials, likely shaped by long-term exposure to heavy metals, such as uranium and mercury. Similar results were obtained by using CARD, which revealed resistomes for the following drugs (percent identity with the corresponding region is shown in parenthesis): fluoroquinolone (22.2%), tetracycline’s (22.2%), cephalosporin (11%), penam (11%), carbapenem (5.6%), cephamycins (5.6%), fosfomycin (5.6%), macrolide (5.6%), monobactam (5.6%), and aminoglycoside (5.6%) (Jia et al., 2017), as shown in Figure 7B. Several other genes that possibly confer antibiotic resistance were also recognized, including antibiotic target protein (Penicillin-binding protein mutations), antibiotic target alteration, antibiotic self-resistance gene, resistance-nodulation-cell division (RND), major facilitator superfamily (MFS), efflux pump complex etc. This overall ARG evaluation of *Serratia* sp. SRS-8-S-2018 indicates that the strain has acquired resistance to multiple antibiotics, most likely exacerbated by exposure to Hg, U and possibly other heavy metal contamination in the SRS soils. These findings are in line with previous body of information on SRS soils, which unequivocally show that the legacy contaminated SRS soils serve as a repository for acquisition of both metal and antibiotic resistances (Stepanauskas et al., 2006; Wright et al., 2006; Thomas et al., 2020). However, this study is focused on *Serratia* spp., which are opportunistic pathogens and brings out the necessity for further focused studies on this aspect rather than a more generalized assessment of the proliferation and abundance of MRGs and ARGs within the SRS soil habitat, as has been the case with most previous studies.

### Phenomic Analysis of *Serratia marcescens* Strain SRS-8-S-2018

**FIGURE 8:**
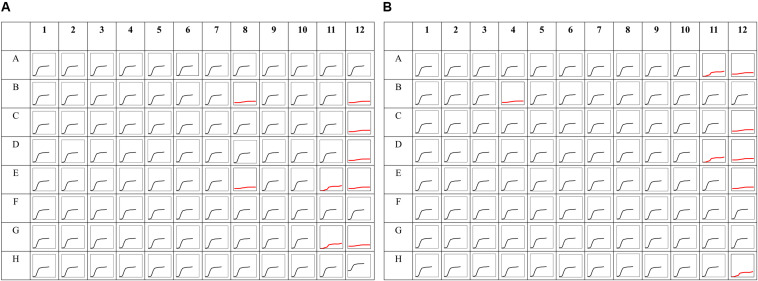
Phenomic analysis of *Serratia* sp. strain SRS-8-S-2018. Shown are **(A)** phenomic analysis profile of *Serratia* sp. SRS-8-S-2018 using the PM MicroPlate^™^ array #11C **(A)** and #12B **(B)**. Each four well block going from left to right contain the same antibiotic with the lowest to the highest concentration. The black curve indicates bacterial resistance, while the red curves indicate bacterial sensitivity to each antibiotic tested. Growth of strain SRS-8-S-2018 was inhibited (displayed as red lines) by the following antibiotics, plate PM-11C positions: B8- Cloxacillin (936 μg/ml), B-12 Lomefloxcin (0.42 μg/ml), C-12 Minocycline (20 μg/ml), D-12 Nafcillin (1060 μg/ml), E-8 Enoxacin (0.9 μg/ml), E-11 Nalidixic Acid (6 μg/ml), G-11 Potassium tellurite (0.3 μg/ml), F-12 Neomycin (1.5 μg/ml), H-12 Ofloxacin (0.17 μg/ml); plate PM12B positions: A-11 Carbenicillin (13 μg/ml), B-4 Oxacillin (1116 μg/ml), C-12 D,L-Serine hydroxamate (844 μg/ml), D-11 Novobiocin (363 μg/ml), E-12 D, L-Serine hydroxamate (103 μg/ml), H-12 Dodecyltrimethyl ammonium bromide (246 μg/ml), respectively.

To validate the functional activity of ARGs in *Serratia* sp. SRS-8-S-2018, phenotype microarray (PM) technique was used, which is a microtiter-plate-based substrate utilization assay (Bochner and Savageau, 1977; Bochner, 1989; Bochner et al., 2001), similar to the Biolog EcoPlates (Borglin et al., 2012). Note that the PM technique, also referred to as phenomics, offers the ability to obtain cellular response of axenic microbiota or environmental communities, as well as screening antimicrobial resistances (Blanco et al., 2016). The results obtained from the PM MicroPlate^™^ analysis are shown in Figures 8A,B. This data is compared in Table 2, showing that only 9 out of 48 antibiotics tested, inhibited or ceased growth, indicating high antibiotic resistance. In fact, 81.25% of the antibiotic/concentration tested did not affect growth, suggesting the broad ARG potential of this strain. The antibiotics and concentrations (as provided by the manufacturer), that affected growth included the following: Cloxacillin (936 μg/ml), Lomefloxcin (0.42 μg/ml), Minocycline (20 μg/ml), Nafcillin (1060 μg/ml), Enoxacin (0.9 μg/ml), Nalidixic Acid (6 μg/ml), Potassium tellurite (0.3 μg/ml), F-12 Neomycin (1.5 μg/ml), Ofloxacin (0.17 μg/ml), Carbenicillin (13 μg/ml), Oxacillin (1,116 μg/ml), D,L-Serine hydroxamate (844 μg/ml), Novobiocin (363 μg/ml) D,L-Serine hydroxamate (103 μg/ml), Dodecyltrimethyl ammonium bromide (246 μg/ml), respectively.

**TABLE 2:** Phenomics analysis using the phenotype PM MicroPlate^™^ array # PM11C and PM12B, which revealed that *Serratia* sp. SRS-8-S-2018 was resistant to 34 and sensitive to 14 tested antimicrobials.

Antibiotic Tested	Sensitive	Intermediate	Resistant
Amikacin			R
Chlortetracycline			R
Lincomycin			R
Amoxicillin			R
Cloxacillin	S		
Lomefloxacin	S		
Bleomycin			R
Colistin			R
Minocycline	S		
Capreomycin			R
Demeclocycline			R
Nafcillin	S		
Cefazolin			R
Enoxacin	S		
Nalidixic Acid	S		
Chloramphenicol			R
Erythromycin			R
Neomycin			R
Ceftriaxone			R
Gentamicin			R
Potassium tellurite	S		
Cephalothin			R
Kanamycin			R
Ofloxacin	S		
Penicillin G			R
Tetracycline			R
Carbenicillin	S		
Oxacillin	S		
Penimepicycline			R
Polymyxin B			R
Paromomycin			R
Vancomycin			R
D,L-serine hydroxamate	S		
Sisomicin			R
Sulfamethazine			R
Novobiocin	S		
2,4-diamino-6,7 diisopropylpteridine			R
Sulfadiazine			R
Benzethonium chloride	S		
Tobramycin			R
Sulfathiazole			R
5-fluoroorotic acid			R
Spectinomycin			R
Sulfamethoxazole			R
L-aspartic-β-hydroxamate			R
Spiramycin			R
Rifampicin			R
Dodecyltrimethyl ammonium bromide	S		

After correlating the findings from the antimicrobial resistance genes (ARGs) analysis and phenotype microarray (PM) antibiotic profile of *Serratia* sp. SRS-8-S-2018, it can be concluded that the *Serratia* sp. SRS-8-S-2018 harbors a suite of ARGs against many antibiotics as well as metal resistant genes (MRGs). The antibiotic profile of *Serratia* sp. SRS-8-S-2018 was also mirrored in the genomic analysis (Figures 7A,B). Biolog PM MicroPlate^™^ array assay on *Serratia* sp. SRS-8-S-2018 yielded an extensive resistance pattern to antibiotics. The development of a broad spectrum of ARGs in strain SRS-8-S-2018 are very likely due to the stress imposed by the long-term contamination of SRS soils with Hg and U, thus presenting with serious public health and ecological risks. Note that Hg resistance is almost invariably found to co-occur with antibiotic resistance, specifically for ampicillin, tetracycline, erythromycin, and penicillin (Ready et al., 2003). Therefore, presence of Hg and U stressors present in the SRS soils pose public health and ecological concerns from the metal driven antibiotic resistome or vice versa.

### Genome-Wide Functional Comparisons Between Environmental and Medical Isolates of *Serratia marcescens*

*Serratia marcescens* represents a recently recognized pathogenic species and hence remains largely understudied. Furthermore, whole genome sequence projects on this pathogen have also only recently become available to the scientific community (Abreo and Altier, 2019), thus limited understanding is available on the genome-wide adaptive traits of *S. marcescens* as a pathogenic agent. Toward this end, this study reveals evolutionary and adaptive traits possessed by an environmental *Serratia* species, and in particular, carriage of heavy metal and antibiotic resistance gene determinants. It is very likely that broad antibiotic resistances are being acquired by microbiomes, such as *Serratia*, in sites that remain historically contaminated with heavy metals, which is a major public health concern. Note that resistance of opportunistic pathogens to one or more antibiotics has been recognized as a global crisis, which needs immediate attention such that a major pathogenic outbreak such as the COVID-19, can be avoided. Even though COVID-19 is a viral disease but some preliminary estimates suggest that 50% of mortalities are occurring from nosocomial secondary infections. Therefore, one broader recommendation from this study is to take immediate action against development of multidrug resistant bacteria and develop strategies should a bacterial pathogen outbreak occur that is similar in magnitude to the ongoing COVID-19, as of this writing.

**FIGURE 9:**
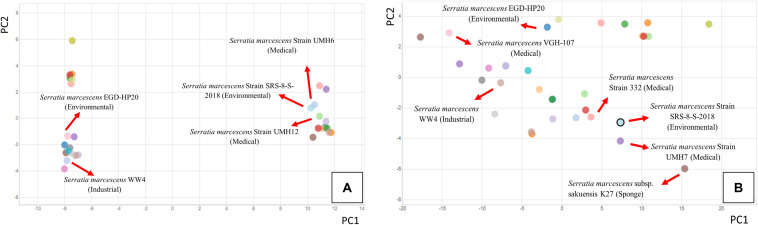
Genome-wide principal-component analysis (PCA) comparison of 31 *S. marcescens* isolates relative to *S. marcescens* strain SRS-8-S-2018 obtained from the Integrated Microbial Genomes (IMG) pipeline. Isolates for this analysis were chosen based on their relevance to environmental/industrial and/or clinical/antibiotic resistance. Shown are **(A)** clustering based on COG profiles for selected genomes and **(B)**, clustering based on KEGG pathway (EC) profiles, respectively.

It is noteworthy that outbreaks related to *S. marcescens* and other *Serratia* species have occurred sporadically since 1950s (Grohskopf et al., 2001; Mahlen, 2011; Yao et al., 2016), mainly in neonatal and cardiac ICUs, orthopedic clinics and dialysis centers. There is a large body of information available on *Serratia* epidemiology and resistance patterns and *S*. *marcescens* strains are typically resistant to all penicillins and susceptible to all carbapenems; a recent study, however, was found resistant to all tested antibiotics (Bertrand and Dowzicky, 2012). However, not much is known on the antimicrobial patterns of environmental *Serratia* members, and this study addresses this gap. More recently, pangenome-based analysis of 49 *S. marcescens* strains from nosocomial and environmental origins as well as different countries, revealed consistently presence of high number of ARGs (Abreo and Altier, 2019), likely due to stress imposed by environmental contaminants, as also suggested in this study on strain SRS-8-S-2018. To obtain a better understanding on the relationship between the environmentally relevant *S. marcescens* strain SRS-8-S-2018 with some other medical/clinically relevant isolates, comparative whole genome analysis was run using the Integrated Microbial Genomes (IMG) system, and results are shown in Figure 9. Specifically, we selected the closest taxonomic relatives of strain SRS-8-S-2018 identified in previous analysis, such as *S*. *marcescens* WW4, *S*. *marcescens* EGD-HP20, along with several strains isolated from bacteremia or clinical settings, such as *S*. *marcescens* UMH12, *S*. *marcescens* 332; a total of 32 *S. marcescens* strains were included in this analysis. Remarkably, strain SRS-8-S-2018 clustered closest to the strains UMH 6 and UMH 12 in a PCA drawn on COG profiles for the selected genomes (Figure 9A). Conversely, strains WW4 and EGD-HP20- the closest taxonomic relatives of strain SRS-8-S-2018, clustered away from the cohort with which strain SRS-8-S-2018 clustered, which were all medically relevant strains. As stated before, COGs represent families of orthologous protein-coding genes and is a reliable assessment of comparing protein phylogeny from microbial genomes (Galperin et al., 2019), and hence, is a functional assessment of a bacteria of interest. This observation was again confirmed when PCA clustering was based on the presence of KEGG pathway (EC) profiles (Figure 9B), which is based on the enzymatically catalyzed network of metabolic pathways and reactions, constructed upon the entirety of all available biochemical information and is not organism-specific. Similar to the COG-based analysis, the closest association of strain SRS-8-S-2018 was with strains UMH7 and 332- many of these strains were active in bacteremia (Anderson et al., 2017). Overall, this is strong evidence that strain SRS-8-S-2018, isolated from a metalliferous contaminated soil environment, is functionally more similar to medically relevant isolates. Overall, this is succinct evidence that the environment is potentially serving as a niche for the development, recruitment and proliferation of pathogenic traits within *Serratia* species, which needs to be evaluated to obtain mitigation strategies and avoidance of possible future pathogenic outbreaks emanating from ecological systems; as is indicative for the origin of the COVID-19 viral pathogen from bats, which is likely the main reservoir for this pathogen (Banerjee et al., 2019; Andersen et al., 2020).

## Data Availability Statement

The datasets presented in this study can be found in online repositories. The names of the repository/repositories and accession number(s) can be found in the section titled “Nucleotide Sequence Accession Number”.

## Author Contributions

SG, MA, AP, and RR designed the experiments. SG, AP, and AC performed the genomic analysis. SG, MA, and RR performed the BIOLOG and phenomic tests and analyzed the results. AC, SG, AP, MA, RR, and RJ drafted the manuscript. All authors contributed to the article and approved the submitted version.

## Conflict of Interest

The authors declare that the research was conducted in the absence of any commercial or financial relationships that could be construed as a potential conflict of interest.

## Footnotes

1 https://www.statnews.com/2020/03/23/antibiotic-resistance-hidden-threat-lurking-behind-covid-19/

2 https://blogs.scientificamerican.com/observations/antibiotic-resistance-could-lead-to-more-covid-19-deaths/

3 https://www.biolog.com/products-portfolio-overview/phenotype-microarrays-for-microbial-cells/

4 https://www.ncbi.nlm.nih.gov/nuccore/VHNF00000000.1

5 https://www.ncbi.nlm.nih.gov/bioproject/PRJNA550097

6 https://www.ncbi.nlm.nih.gov/biosample/SAMN12107717

7 https://doi.org/10.6084/m9.figshare.6253847.v2
