# Supplementary material for: Is Long-Term Heavy Metal Exposure Driving Carriage of Antibiotic Resistance in Environmental Opportunistic Pathogens: A Comprehensive Phenomic and Genomic Assessment Using Serratia sp. SRS-8-S-2018
Source: Front Microbiol. 2020 Aug 20;11:1923. doi: 10.3389/fmicb.2020.01923 (PMC7468404; doi:10.3389/fmicb.2020.01923)
Supplement: Supplementary file 1 [file Table_1.DOCX]

**Table SI-1:** Listed are 156 candidate genes that likely have prophage remnants within the genome of *Serratia* sp. strain SRS-8-S-2018 identified using the prophage hunter pipeline.

| **Candidate ID** | **Sequence ID** | **Start** | **End** | **Length** | **Category** | **Score** | **Closest phage** | **Gene number** |
| --- | --- | --- | --- | --- | --- | --- | --- | --- |
| Candidate_133 | MA8_S11_L001_R1_001_(paired)_trimmed_(paired)_contig_6 | 135091 | 203199 | 68109 | Inactive | 0.12 | Salmonella phage FSL SP-004 | 72 |
| Candidate_33 | MA8_S11_L001_R1_001_(paired)_trimmed_(paired)_contig_15 | 48540 | 101004 | 52465 | Inactive | 0.45 | Salmonella phage 29485 | 70 |
| Candidate_78 | MA8_S11_L001_R1_001_(paired)_trimmed_(paired)_contig_32 | 97786 | 145326 | 47541 | Inactive | 0.01 | Nocardia phage NBR1 | 50 |
| Candidate_18 | MA8_S11_L001_R1_001_(paired)_trimmed_(paired)_contig_12 | 9529 | 56590 | 47062 | Inactive | 0.02 | Edwardsiella phage PEi26 | 48 |
| Candidate_75 | MA8_S11_L001_R1_001_(paired)_trimmed_(paired)_contig_30 | 73683 | 112410 | 38728 | Inactive | 0.02 | Klebsiella phage 4 LV-2017 | 40 |
| Candidate_39 | MA8_S11_L001_R1_001_(paired)_trimmed_(paired)_contig_17 | 62555 | 98056 | 35502 | Inactive | 0.11 | Bacillus phage BCU4 | 39 |
| Candidate_3 | MA8_S11_L001_R1_001_(paired)_trimmed_(paired)_contig_1 | 51940 | 95824 | 43885 | Inactive | 0 | Streptococcus phage phiZJ20091101-2 | 37 |
| Candidate_24 | MA8_S11_L001_R1_001_(paired)_trimmed_(paired)_contig_14 | 63583 | 101281 | 37699 | Inactive | 0.02 | Stx2-converting phage 1717 | 35 |
| Candidate_49 | MA8_S11_L001_R1_001_(paired)_trimmed_(paired)_contig_20 | 16244 | 49947 | 33704 | Inactive | 0.01 | Moraxella phage Mcat6 | 34 |
| Candidate_79 | MA8_S11_L001_R1_001_(paired)_trimmed_(paired)_contig_32 | 136379 | 172343 | 35965 | Inactive | 0.01 | Skermania phage SPI1 | 33 |
| Candidate_91 | MA8_S11_L001_R1_001_(paired)_trimmed_(paired)_contig_35 | 190244 | 215969 | 25726 | Inactive | 0.02 | Morganella phage IME1369_01 | 33 |
| Candidate_92 | MA8_S11_L001_R1_001_(paired)_trimmed_(paired)_contig_36 | 4084 | 35721 | 31638 | Inactive | 0.02 | Pseudomonas phage phi3 | 33 |
| Candidate_148 | MA8_S11_L001_R1_001_(paired)_trimmed_(paired)_contig_73 | 936 | 22919 | 21984 | Inactive | 0.08 | Mycobacterium phage Terror | 33 |
| Candidate_156 | MA8_S11_L001_R1_001_(paired)_trimmed_(paired)_contig_9 | 68445 | 95569 | 27125 | Inactive | 0.18 | Streptomyces phage Picard | 33 |
| Candidate_4 | MA8_S11_L001_R1_001_(paired)_trimmed_(paired)_contig_1 | 107759 | 150669 | 42911 | Inactive | 0 | Pseudomonas phage Noxifer | 32 |
| Candidate_77 | MA8_S11_L001_R1_001_(paired)_trimmed_(paired)_contig_32 | 11157 | 42797 | 31641 | Inactive | 0.01 | Salmonella phage SJ46 | 32 |
| Candidate_35 | MA8_S11_L001_R1_001_(paired)_trimmed_(paired)_contig_16 | 34066 | 68142 | 34077 | Inactive | 0.01 | Pseudomonas phage pf16 | 31 |
| Candidate_56 | MA8_S11_L001_R1_001_(paired)_trimmed_(paired)_contig_21 | 60443 | 94000 | 33558 | Inactive | 0.02 | Mycobacterium phage Mufasa | 31 |
| Candidate_127 | MA8_S11_L001_R1_001_(paired)_trimmed_(paired)_contig_52 | 79046 | 112228 | 33183 | Inactive | 0.01 | Salmonella phage SEN22 | 31 |
| Candidate_26 | MA8_S11_L001_R1_001_(paired)_trimmed_(paired)_contig_14 | 101934 | 138084 | 36151 | Inactive | 0.01 | Streptomyces phage SqueakyClean | 30 |
| Candidate_32 | MA8_S11_L001_R1_001_(paired)_trimmed_(paired)_contig_15 | 16733 | 42703 | 25971 | Inactive | 0.02 | Skermania phage SPI1 | 30 |
| Candidate_47 | MA8_S11_L001_R1_001_(paired)_trimmed_(paired)_contig_2 | 66630 | 93225 | 26596 | Inactive | 0.05 | Klebsiella phage 2b LV-2017 | 30 |
| Candidate_89 | MA8_S11_L001_R1_001_(paired)_trimmed_(paired)_contig_35 | 166723 | 198293 | 31571 | Inactive | 0.01 | Morganella phage IME1369_01 | 30 |
| Candidate_38 | MA8_S11_L001_R1_001_(paired)_trimmed_(paired)_contig_17 | 42273 | 79790 | 37518 | Inactive | 0.04 | Bacillus phage BCU4 | 29 |
| Candidate_93 | MA8_S11_L001_R1_001_(paired)_trimmed_(paired)_contig_36 | 63053 | 93101 | 30049 | Inactive | 0.02 | Bordetella phage BIP-1 | 29 |
| Candidate_58 | MA8_S11_L001_R1_001_(paired)_trimmed_(paired)_contig_22 | 22909 | 51123 | 28215 | Inactive | 0 | Bacillus phage proCM3 | 28 |
| Candidate_96 | MA8_S11_L001_R1_001_(paired)_trimmed_(paired)_contig_37 | 36437 | 65482 | 29046 | Inactive | 0.02 | Mycobacterium phage ILeeKay | 28 |
| Candidate_106 | MA8_S11_L001_R1_001_(paired)_trimmed_(paired)_contig_40 | 1859 | 39505 | 37647 | Inactive | 0 | Morganella phage vB_MmoM_MP1 | 28 |
| Candidate_137 | MA8_S11_L001_R1_001_(paired)_trimmed_(paired)_contig_61 | 7866 | 38156 | 30291 | Inactive | 0.01 | Mycobacterium phage Phabba | 28 |
| Candidate_2 | MA8_S11_L001_R1_001_(paired)_trimmed_(paired)_contig_1 | 14342 | 43373 | 29032 | Inactive | 0.02 | N/A | 27 |
| Candidate_45 | MA8_S11_L001_R1_001_(paired)_trimmed_(paired)_contig_2 | 250 | 30882 | 30633 | Inactive | 0 | Mycobacterium phage Reprobate | 27 |
| Candidate_51 | MA8_S11_L001_R1_001_(paired)_trimmed_(paired)_contig_20 | 75907 | 101589 | 25683 | Inactive | 0.01 | N/A | 27 |
| Candidate_88 | MA8_S11_L001_R1_001_(paired)_trimmed_(paired)_contig_35 | 140627 | 168196 | 27570 | Inactive | 0.01 | Klebsiella phage 6 LV-2017 | 27 |
| Candidate_1 | MA8_S11_L001_R1_001_(paired)_trimmed_(paired)_contig_1 | 2587 | 28403 | 25817 | Inactive | 0.06 | Mycobacterium phage Kumao | 26 |
| Candidate_41 | MA8_S11_L001_R1_001_(paired)_trimmed_(paired)_contig_17 | 147848 | 174148 | 26301 | Inactive | 0.11 | Streptococcus phage phi1207.3 | 26 |
| Candidate_54 | MA8_S11_L001_R1_001_(paired)_trimmed_(paired)_contig_21 | 29500 | 60060 | 30561 | Inactive | 0 | Mycobacterium phage Shandong1 | 26 |
| Candidate_83 | MA8_S11_L001_R1_001_(paired)_trimmed_(paired)_contig_35 | 48847 | 75241 | 26395 | Inactive | 0.01 | Caulobacter phage Ccr34 | 26 |
| Candidate_85 | MA8_S11_L001_R1_001_(paired)_trimmed_(paired)_contig_35 | 91129 | 117731 | 26603 | Inactive | 0.1 | Enterobacter phage PG7 | 26 |
| Candidate_86 | MA8_S11_L001_R1_001_(paired)_trimmed_(paired)_contig_35 | 91283 | 117985 | 26703 | Inactive | 0.1 | Enterobacter phage PG7 | 26 |
| Candidate_99 | MA8_S11_L001_R1_001_(paired)_trimmed_(paired)_contig_39 | 93392 | 119275 | 25884 | Inactive | 0.01 | Morganella phage IME1369_01 | 26 |
| Candidate_108 | MA8_S11_L001_R1_001_(paired)_trimmed_(paired)_contig_42 | 16578 | 51103 | 34526 | Inactive | 0.01 | Escherichia phage D6 | 26 |
| Candidate_115 | MA8_S11_L001_R1_001_(paired)_trimmed_(paired)_contig_46 | 51823 | 77069 | 25247 | Inactive | 0.19 | Cronobacter phage CR3 | 26 |
| Candidate_17 | MA8_S11_L001_R1_001_(paired)_trimmed_(paired)_contig_12 | 5709 | 30127 | 24419 | Inactive | 0.03 | Xylella phage Salvo | 25 |
| Candidate_80 | MA8_S11_L001_R1_001_(paired)_trimmed_(paired)_contig_32 | 159926 | 191865 | 31940 | Inactive | 0 | Salmonella phage Melville | 25 |
| Candidate_81 | MA8_S11_L001_R1_001_(paired)_trimmed_(paired)_contig_34 | 6593 | 36766 | 30174 | Inactive | 0.01 | Morganella phage IME1369_03 | 25 |
| Candidate_84 | MA8_S11_L001_R1_001_(paired)_trimmed_(paired)_contig_35 | 73542 | 104066 | 30525 | Inactive | 0.03 | Serratia phage X20 | 25 |
| Candidate_110 | MA8_S11_L001_R1_001_(paired)_trimmed_(paired)_contig_43 | 4296 | 32586 | 28291 | Inactive | 0.02 | Ralstonia phage RSB3 | 25 |
| Candidate_126 | MA8_S11_L001_R1_001_(paired)_trimmed_(paired)_contig_52 | 40590 | 66465 | 25876 | Inactive | 0.03 | Cronobacter phage S13 | 25 |
| Candidate_12 | MA8_S11_L001_R1_001_(paired)_trimmed_(paired)_contig_10 | 93668 | 115831 | 22164 | Inactive | 0.01 | N/A | 24 |
| Candidate_25 | MA8_S11_L001_R1_001_(paired)_trimmed_(paired)_contig_14 | 82190 | 109127 | 26938 | Inactive | 0 | Stx2-converting phage 1717 | 24 |
| Candidate_104 | MA8_S11_L001_R1_001_(paired)_trimmed_(paired)_contig_4 | 70453 | 94754 | 24302 | Inactive | 0 | N/A | 24 |
| Candidate_102 | MA8_S11_L001_R1_001_(paired)_trimmed_(paired)_contig_4 | 7743 | 29241 | 21499 | Inactive | 0.02 | Klebsiella phage 1513 | 23 |
| Candidate_125 | MA8_S11_L001_R1_001_(paired)_trimmed_(paired)_contig_52 | 4885 | 27707 | 22823 | Inactive | 0.01 | Pseudomonas phage YMC11/11/R1836 | 23 |
| Candidate_9 | MA8_S11_L001_R1_001_(paired)_trimmed_(paired)_contig_10 | 29961 | 56780 | 26820 | Inactive | 0.02 | Rhodovulum phage vB_RhkS_P1 | 22 |
| Candidate_30 | MA8_S11_L001_R1_001_(paired)_trimmed_(paired)_contig_14 | 220453 | 242191 | 21739 | Inactive | 0 | Moraxella phage Mcat18 | 22 |
| Candidate_37 | MA8_S11_L001_R1_001_(paired)_trimmed_(paired)_contig_17 | 17646 | 43878 | 26233 | Inactive | 0 | Erwinia phage vB_EamM_Yoloswag | 22 |
| Candidate_55 | MA8_S11_L001_R1_001_(paired)_trimmed_(paired)_contig_21 | 48721 | 76785 | 28065 | Inactive | 0.01 | Mycobacterium phage Mufasa | 22 |
| Candidate_65 | MA8_S11_L001_R1_001_(paired)_trimmed_(paired)_contig_27 | 29183 | 58925 | 29743 | Inactive | 0.01 | Aeromonas phage PX29 | 22 |
| Candidate_67 | MA8_S11_L001_R1_001_(paired)_trimmed_(paired)_contig_27 | 78083 | 108278 | 30196 | Inactive | 0.02 | Escherichia phage ESCO5 | 22 |
| Candidate_107 | MA8_S11_L001_R1_001_(paired)_trimmed_(paired)_contig_40 | 55146 | 88267 | 33122 | Inactive | 0 | Pseudomonas phage PaMx11 | 22 |
| Candidate_142 | MA8_S11_L001_R1_001_(paired)_trimmed_(paired)_contig_66 | 4583 | 31756 | 27174 | Inactive | 0.01 | Streptomyces phage Diane | 22 |
| Candidate_155 | MA8_S11_L001_R1_001_(paired)_trimmed_(paired)_contig_9 | 53413 | 71350 | 17938 | Inactive | 0.13 | Propionibacterium phage Keiki | 22 |
| Candidate_14 | MA8_S11_L001_R1_001_(paired)_trimmed_(paired)_contig_11 | 32563 | 53833 | 21271 | Inactive | 0.01 | Enterobacteria phage YYZ-2008 | 21 |
| Candidate_21 | MA8_S11_L001_R1_001_(paired)_trimmed_(paired)_contig_13 | 10448 | 32994 | 22547 | Inactive | 0.01 | Mycobacterium phage Zemanar | 21 |
| Candidate_46 | MA8_S11_L001_R1_001_(paired)_trimmed_(paired)_contig_2 | 40090 | 63839 | 23750 | Inactive | 0.1 | Agrobacterium phage Atu_ph07 | 21 |
| Candidate_60 | MA8_S11_L001_R1_001_(paired)_trimmed_(paired)_contig_23 | 5478 | 25388 | 19911 | Inactive | 0.01 | Klebsiella phage 2b LV-2017 | 21 |
| Candidate_70 | MA8_S11_L001_R1_001_(paired)_trimmed_(paired)_contig_29 | 20192 | 42255 | 22064 | Inactive | 0.01 | Aeromonas phage phiARM81mr | 21 |
| Candidate_105 | MA8_S11_L001_R1_001_(paired)_trimmed_(paired)_contig_4 | 90943 | 114312 | 23370 | Inactive | 0.01 | Gordonia phage Splinter | 21 |
| Candidate_117 | MA8_S11_L001_R1_001_(paired)_trimmed_(paired)_contig_49 | 8177 | 27084 | 18908 | Inactive | 0.02 | Stx2-converting phage 1717 | 21 |
| Candidate_6 | MA8_S11_L001_R1_001_(paired)_trimmed_(paired)_contig_1 | 151390 | 176494 | 25105 | Inactive | 0.01 | Tsukamurella phage TPA2 | 20 |
| Candidate_82 | MA8_S11_L001_R1_001_(paired)_trimmed_(paired)_contig_35 | 18681 | 43783 | 25103 | Inactive | 0.01 | Mycobacterium phage LizLemon | 20 |
| Candidate_87 | MA8_S11_L001_R1_001_(paired)_trimmed_(paired)_contig_35 | 119704 | 140883 | 21180 | Inactive | 0.02 | Xylella phage Sano | 20 |
| Candidate_97 | MA8_S11_L001_R1_001_(paired)_trimmed_(paired)_contig_39 | 60685 | 79281 | 18597 | Inactive | 0.02 | Pectobacterium phage ZF40 | 20 |
| Candidate_129 | MA8_S11_L001_R1_001_(paired)_trimmed_(paired)_contig_53 | 47893 | 69337 | 21445 | Inactive | 0.03 | Planktothrix phage PaV-LD | 20 |
| Candidate_144 | MA8_S11_L001_R1_001_(paired)_trimmed_(paired)_contig_68 | 5588 | 26898 | 21311 | Inactive | 0.01 | Mycobacterium phage Bo4 | 20 |
| Candidate_150 | MA8_S11_L001_R1_001_(paired)_trimmed_(paired)_contig_81 | 5828 | 25904 | 20077 | Inactive | 0.03 | Edwardsiella phage GF-2 | 20 |
| Candidate_5 | MA8_S11_L001_R1_001_(paired)_trimmed_(paired)_contig_1 | 124786 | 148869 | 24084 | Inactive | 0.01 | Pseudomonas phage Noxifer | 19 |
| Candidate_50 | MA8_S11_L001_R1_001_(paired)_trimmed_(paired)_contig_20 | 44903 | 66044 | 21142 | Inactive | 0.01 | Salmonella phage SPC32N | 19 |
| Candidate_62 | MA8_S11_L001_R1_001_(paired)_trimmed_(paired)_contig_23 | 25741 | 50510 | 24770 | Inactive | 0.01 | Rhizobium phage vB_RleM_PPF1 | 19 |
| Candidate_64 | MA8_S11_L001_R1_001_(paired)_trimmed_(paired)_contig_26 | 596 | 12396 | 11801 | Inactive | 0.36 | Stx2-converting phage Stx2a_WGPS2 | 19 |
| Candidate_132 | MA8_S11_L001_R1_001_(paired)_trimmed_(paired)_contig_58 | 39685 | 61865 | 22181 | Inactive | 0 | Ralstonia phage RSL2 | 19 |
| Candidate_143 | MA8_S11_L001_R1_001_(paired)_trimmed_(paired)_contig_67 | 4276 | 28668 | 24393 | Inactive | 0 | Escherichia phage phAPEC8 | 19 |
| Candidate_10 | MA8_S11_L001_R1_001_(paired)_trimmed_(paired)_contig_10 | 51895 | 72516 | 20622 | Inactive | 0.01 | Gordonia phage OneUp | 18 |
| Candidate_11 | MA8_S11_L001_R1_001_(paired)_trimmed_(paired)_contig_10 | 82153 | 99916 | 17764 | Inactive | 0.01 | Moraxella phage Mcat7 | 18 |
| Candidate_31 | MA8_S11_L001_R1_001_(paired)_trimmed_(paired)_contig_15 | 4706 | 25869 | 21164 | Inactive | 0.01 | Pseudomonas phage JBD69 | 18 |
| Candidate_43 | MA8_S11_L001_R1_001_(paired)_trimmed_(paired)_contig_18 | 50279 | 68608 | 18330 | Inactive | 0.04 | Mycobacterium phage Wiks | 18 |
| Candidate_63 | MA8_S11_L001_R1_001_(paired)_trimmed_(paired)_contig_26 | 493 | 12253 | 11761 | Inactive | 0.41 | Cyanophage Syn2 | 18 |
| Candidate_69 | MA8_S11_L001_R1_001_(paired)_trimmed_(paired)_contig_28 | 2212 | 25069 | 22858 | Inactive | 0.06 | Stx2-converting phage Stx2a_WGPS2 | 18 |
| Candidate_130 | MA8_S11_L001_R1_001_(paired)_trimmed_(paired)_contig_56 | 664 | 18326 | 17663 | Inactive | 0.1 | Streptomyces phage YDN12 | 18 |
| Candidate_134 | MA8_S11_L001_R1_001_(paired)_trimmed_(paired)_contig_60 | 12719 | 35619 | 22901 | Ambiguous | 0.5 | Salmonella phage SJ46 | 18 |
| Candidate_139 | MA8_S11_L001_R1_001_(paired)_trimmed_(paired)_contig_63 | 21628 | 42458 | 20831 | Inactive | 0.01 | Halorubrum phage CGphi46 | 18 |
| Candidate_140 | MA8_S11_L001_R1_001_(paired)_trimmed_(paired)_contig_63 | 21795 | 42562 | 20768 | Inactive | 0.01 | Halorubrum phage CGphi46 | 18 |
| Candidate_145 | MA8_S11_L001_R1_001_(paired)_trimmed_(paired)_contig_68 | 10629 | 30512 | 19884 | Inactive | 0.01 | Gordonia phage Jeanie | 18 |
| Candidate_151 | MA8_S11_L001_R1_001_(paired)_trimmed_(paired)_contig_81 | 13345 | 33610 | 20266 | Inactive | 0.03 | Thiobacimonas phage vB_ThpS-P1 | 18 |
| Candidate_57 | MA8_S11_L001_R1_001_(paired)_trimmed_(paired)_contig_22 | 7552 | 30613 | 23062 | Inactive | 0 | Moraxella phage Mcat3 | 17 |
| Candidate_59 | MA8_S11_L001_R1_001_(paired)_trimmed_(paired)_contig_22 | 47399 | 70929 | 23531 | Inactive | 0 | Haemophilus phage SuMu | 17 |
| Candidate_94 | MA8_S11_L001_R1_001_(paired)_trimmed_(paired)_contig_36 | 84326 | 107309 | 22984 | Inactive | 0.01 | Gordonia phage Lysidious | 17 |
| Candidate_95 | MA8_S11_L001_R1_001_(paired)_trimmed_(paired)_contig_37 | 10492 | 30710 | 20219 | Inactive | 0.01 | Erwinia phage vB_EamM_Yoloswag | 17 |
| Candidate_138 | MA8_S11_L001_R1_001_(paired)_trimmed_(paired)_contig_61 | 34962 | 52361 | 17400 | Inactive | 0.01 | Mycobacterium phage Phabba | 17 |
| Candidate_154 | MA8_S11_L001_R1_001_(paired)_trimmed_(paired)_contig_9 | 26657 | 45830 | 19174 | Inactive | 0 | Salmonella phage SP069 | 17 |
| Candidate_34 | MA8_S11_L001_R1_001_(paired)_trimmed_(paired)_contig_16 | 29117 | 45482 | 16366 | Inactive | 0.01 | Saccharomonospora phage PIS 136 | 16 |
| Candidate_53 | MA8_S11_L001_R1_001_(paired)_trimmed_(paired)_contig_21 | 6780 | 30286 | 23507 | Inactive | 0.01 | Geobacillus virus E2 | 16 |
| Candidate_74 | MA8_S11_L001_R1_001_(paired)_trimmed_(paired)_contig_30 | 13818 | 44383 | 30566 | Inactive | 0 | N/A | 16 |
| Candidate_90 | MA8_S11_L001_R1_001_(paired)_trimmed_(paired)_contig_35 | 190287 | 207606 | 17320 | Inactive | 0.01 | Arthrobacter phage PrincessTrina | 16 |
| Candidate_122 | MA8_S11_L001_R1_001_(paired)_trimmed_(paired)_contig_5 | 151793 | 171388 | 19596 | Inactive | 0.05 | Ralstonia phage RSY1 | 16 |
| Candidate_68 | MA8_S11_L001_R1_001_(paired)_trimmed_(paired)_contig_27 | 123338 | 139120 | 15783 | Inactive | 0.33 | Arthrobacter phage Piccoletto | 15 |
| Candidate_71 | MA8_S11_L001_R1_001_(paired)_trimmed_(paired)_contig_29 | 69938 | 85662 | 15725 | Inactive | 0 | Burkholderia phage AH2 | 15 |
| Candidate_114 | MA8_S11_L001_R1_001_(paired)_trimmed_(paired)_contig_46 | 20229 | 36143 | 15915 | Inactive | 0.36 | N/A | 15 |
| Candidate_131 | MA8_S11_L001_R1_001_(paired)_trimmed_(paired)_contig_58 | 8415 | 26394 | 17980 | Inactive | 0.07 | Enterobacteria phage P88 | 15 |
| Candidate_135 | MA8_S11_L001_R1_001_(paired)_trimmed_(paired)_contig_60 | 28719 | 43812 | 15094 | Inactive | 0.29 | Salmonella phage SJ46 | 15 |
| Candidate_153 | MA8_S11_L001_R1_001_(paired)_trimmed_(paired)_contig_87 | 3278 | 18563 | 15286 | Inactive | 0.01 | N/A | 15 |
| Candidate_66 | MA8_S11_L001_R1_001_(paired)_trimmed_(paired)_contig_27 | 50343 | 70582 | 20240 | Inactive | 0.01 | Gordonia phage GMA6 | 14 |
| Candidate_100 | MA8_S11_L001_R1_001_(paired)_trimmed_(paired)_contig_39 | 132588 | 148436 | 15849 | Inactive | 0.02 | Mycobacterium phage AlanGrant | 14 |
| Candidate_23 | MA8_S11_L001_R1_001_(paired)_trimmed_(paired)_contig_14 | 23189 | 39205 | 16017 | Inactive | 0.01 | Burkholderia phage KS5 | 13 |
| Candidate_27 | MA8_S11_L001_R1_001_(paired)_trimmed_(paired)_contig_14 | 120471 | 137911 | 17441 | Inactive | 0.02 | Mycobacterium phage LastHope | 13 |
| Candidate_29 | MA8_S11_L001_R1_001_(paired)_trimmed_(paired)_contig_14 | 164423 | 183655 | 19233 | Inactive | 0 | Salmonella phage SSU5 | 13 |
| Candidate_61 | MA8_S11_L001_R1_001_(paired)_trimmed_(paired)_contig_23 | 16252 | 33183 | 16932 | Inactive | 0.02 | Mycobacterium phage Pari | 13 |
| Candidate_146 | MA8_S11_L001_R1_001_(paired)_trimmed_(paired)_contig_7 | 19300 | 33512 | 14213 | Inactive | 0.07 | Escherichia phage Rac-SA53 | 13 |
| Candidate_13 | MA8_S11_L001_R1_001_(paired)_trimmed_(paired)_contig_11 | 7433 | 21149 | 13717 | Inactive | 0 | Pseudomonas phage F10 | 12 |
| Candidate_16 | MA8_S11_L001_R1_001_(paired)_trimmed_(paired)_contig_12 | 123 | 13274 | 13152 | Inactive | 0 | Xylella phage Salvo | 12 |
| Candidate_19 | MA8_S11_L001_R1_001_(paired)_trimmed_(paired)_contig_12 | 63014 | 76487 | 13474 | Inactive | 0.05 | Salmonella phage SSE121 | 12 |
| Candidate_44 | MA8_S11_L001_R1_001_(paired)_trimmed_(paired)_contig_19 | 12315 | 28076 | 15762 | Inactive | 0.02 | Mycobacterium phage Apocalypse | 12 |
| Candidate_52 | MA8_S11_L001_R1_001_(paired)_trimmed_(paired)_contig_21 | 144 | 19579 | 19436 | Inactive | 0 | Geobacillus virus E2 | 12 |
| Candidate_73 | MA8_S11_L001_R1_001_(paired)_trimmed_(paired)_contig_30 | 3925 | 33094 | 29170 | Inactive | 0 | N/A | 12 |
| Candidate_76 | MA8_S11_L001_R1_001_(paired)_trimmed_(paired)_contig_32 | 18638 | 29761 | 11124 | Inactive | 0.03 | Salmonella phage SJ46 | 12 |
| Candidate_109 | MA8_S11_L001_R1_001_(paired)_trimmed_(paired)_contig_43 | 900 | 13625 | 12726 | Inactive | 0.03 | N/A | 12 |
| Candidate_112 | MA8_S11_L001_R1_001_(paired)_trimmed_(paired)_contig_44 | 50452 | 61661 | 11210 | Inactive | 0.02 | Enterobacteria phage VT2phi_272 | 12 |
| Candidate_119 | MA8_S11_L001_R1_001_(paired)_trimmed_(paired)_contig_5 | 14159 | 27919 | 13761 | Inactive | 0 | Caulobacter phage Ccr5 | 12 |
| Candidate_8 | MA8_S11_L001_R1_001_(paired)_trimmed_(paired)_contig_10 | 4865 | 27645 | 22781 | Inactive | 0 | Streptomyces phage Ididsumtinwong | 11 |
| Candidate_22 | MA8_S11_L001_R1_001_(paired)_trimmed_(paired)_contig_14 | 14594 | 31691 | 17098 | Inactive | 0.1 | Ralstonia phage RSY1 | 11 |
| Candidate_42 | MA8_S11_L001_R1_001_(paired)_trimmed_(paired)_contig_18 | 4320 | 18967 | 14648 | Inactive | 0.01 | Synechococcus phage S-WAM1 | 11 |
| Candidate_48 | MA8_S11_L001_R1_001_(paired)_trimmed_(paired)_contig_20 | 1210 | 15449 | 14240 | Inactive | 0 | Proteus phage vB_PmiM_Pm5461 | 11 |
| Candidate_98 | MA8_S11_L001_R1_001_(paired)_trimmed_(paired)_contig_39 | 78247 | 88994 | 10748 | Inactive | 0.15 | Pectobacterium phage ZF40 | 11 |
| Candidate_116 | MA8_S11_L001_R1_001_(paired)_trimmed_(paired)_contig_47 | 33059 | 45719 | 12661 | Inactive | 0.28 | Enterobacteria phage P4 | 11 |
| Candidate_120 | MA8_S11_L001_R1_001_(paired)_trimmed_(paired)_contig_5 | 76111 | 92724 | 16614 | Inactive | 0.01 | Escherichia coli O157 typing phage 10 | 11 |
| Candidate_121 | MA8_S11_L001_R1_001_(paired)_trimmed_(paired)_contig_5 | 131223 | 147375 | 16153 | Inactive | 0 | Gordonia phage Vivi2 | 11 |
| Candidate_141 | MA8_S11_L001_R1_001_(paired)_trimmed_(paired)_contig_64 | 6066 | 16831 | 10766 | Inactive | 0.02 | Gordonia phage Bantam | 11 |
| Candidate_147 | MA8_S11_L001_R1_001_(paired)_trimmed_(paired)_contig_72 | 124 | 12630 | 12507 | Inactive | 0.02 | Erwinia phage EtG | 11 |
| Candidate_152 | MA8_S11_L001_R1_001_(paired)_trimmed_(paired)_contig_84 | 356 | 12365 | 12010 | Inactive | 0.31 | Ochrobactrum phage POA1180 | 11 |
| Candidate_40 | MA8_S11_L001_R1_001_(paired)_trimmed_(paired)_contig_17 | 130124 | 146550 | 16427 | Inactive | 0 | Roseobacter phage DSS3P8 | 10 |
| Candidate_72 | MA8_S11_L001_R1_001_(paired)_trimmed_(paired)_contig_30 | 547 | 28067 | 27521 | Inactive | 0 | N/A | 10 |
| Candidate_111 | MA8_S11_L001_R1_001_(paired)_trimmed_(paired)_contig_43 | 106852 | 117838 | 10987 | Inactive | 0.05 | Mycobacterium phage CASbig | 10 |
| Candidate_124 | MA8_S11_L001_R1_001_(paired)_trimmed_(paired)_contig_52 | 542 | 11610 | 11069 | Inactive | 0.01 | Pseudomonas phage PaBG | 10 |
| Candidate_128 | MA8_S11_L001_R1_001_(paired)_trimmed_(paired)_contig_53 | 2748 | 17092 | 14345 | Inactive | 0.01 | Mycobacterium phage Cooper | 10 |
| Candidate_7 | MA8_S11_L001_R1_001_(paired)_trimmed_(paired)_contig_10 | 317 | 14886 | 14570 | Inactive | 0 | Rhodococcus phage E3 | 9 |
| Candidate_20 | MA8_S11_L001_R1_001_(paired)_trimmed_(paired)_contig_13 | 615 | 11785 | 11171 | Inactive | 0.01 | Caulobacter phage Seuss | 9 |
| Candidate_28 | MA8_S11_L001_R1_001_(paired)_trimmed_(paired)_contig_14 | 136925 | 147044 | 10120 | Inactive | 0 | Streptomyces phage Xkcd426 | 9 |
| Candidate_113 | MA8_S11_L001_R1_001_(paired)_trimmed_(paired)_contig_45 | 1720 | 14972 | 13253 | Inactive | 0 | N/A | 9 |
| Candidate_123 | MA8_S11_L001_R1_001_(paired)_trimmed_(paired)_contig_50 | 1834 | 12974 | 11141 | Inactive | 0.05 | Rhizobium phage vB_RleS_L338C | 9 |
| Candidate_103 | MA8_S11_L001_R1_001_(paired)_trimmed_(paired)_contig_4 | 50640 | 62917 | 12278 | Inactive | 0 | Caulobacter phage Sansa | 7 |
