# Supplementary material for: Is Long-Term Heavy Metal Exposure Driving Carriage of Antibiotic Resistance in Environmental Opportunistic Pathogens: A Comprehensive Phenomic and Genomic Assessment Using Serratia sp. SRS-8-S-2018
Source: Front Microbiol. 2020 Aug 20;11:1923. doi: 10.3389/fmicb.2020.01923 (PMC7468404; doi:10.3389/fmicb.2020.01923)

**Fig. SI-1.** Shown are comparisons of average nucleotide identity (ANI) scores of strain SRS-8-S-2018 relative to 15 other sequenced *Serratia* species.


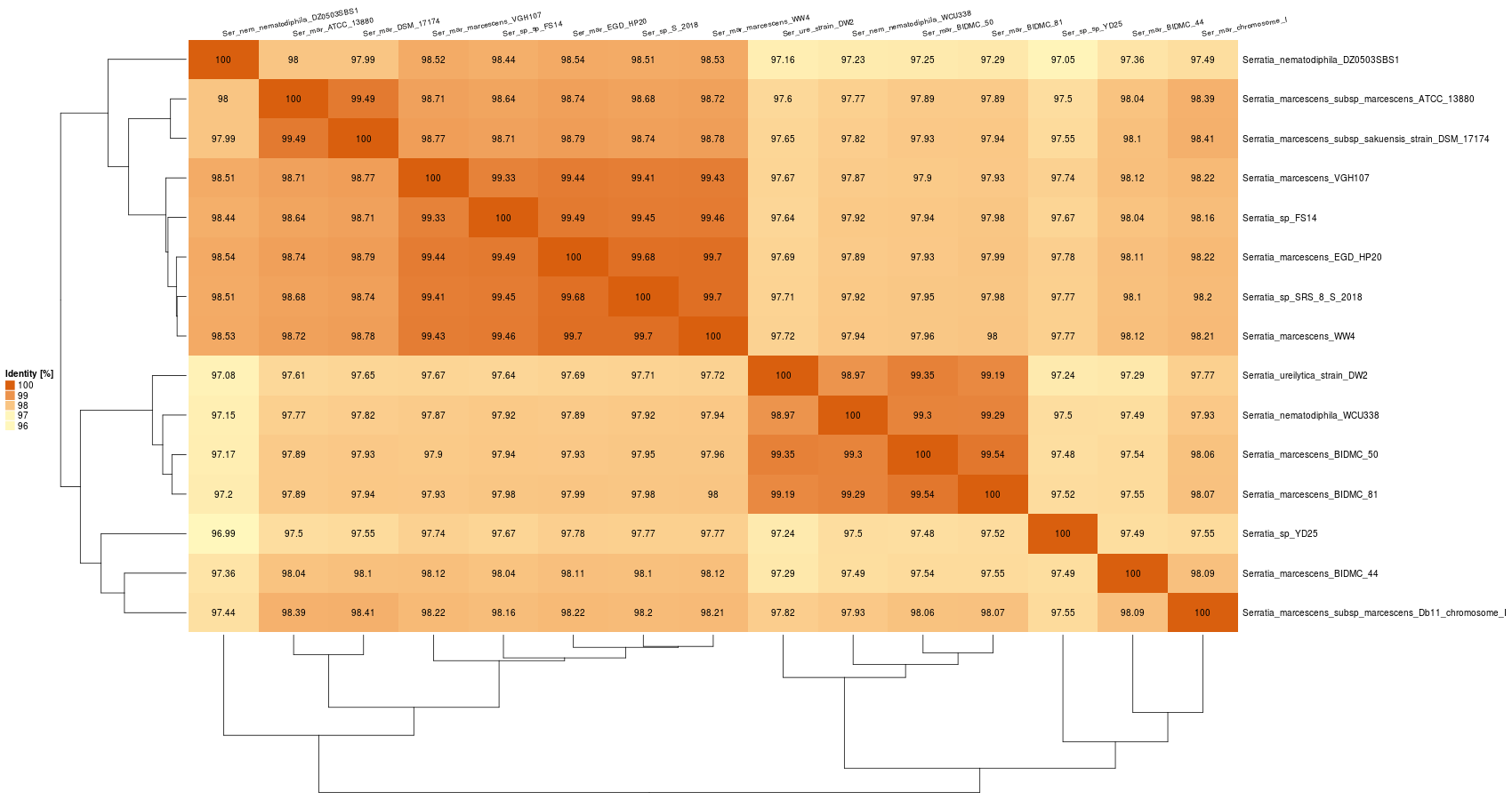

Supplement: Supplementary file 2 [file Table_2.DOCX]
