# Supplementary material for: Is Long-Term Heavy Metal Exposure Driving Carriage of Antibiotic Resistance in Environmental Opportunistic Pathogens: A Comprehensive Phenomic and Genomic Assessment Using Serratia sp. SRS-8-S-2018
Source: Front Microbiol. 2020 Aug 20;11:1923. doi: 10.3389/fmicb.2020.01923 (PMC7468404; doi:10.3389/fmicb.2020.01923)
Supplement: Supplementary file 3 [file Table_3.DOCX]

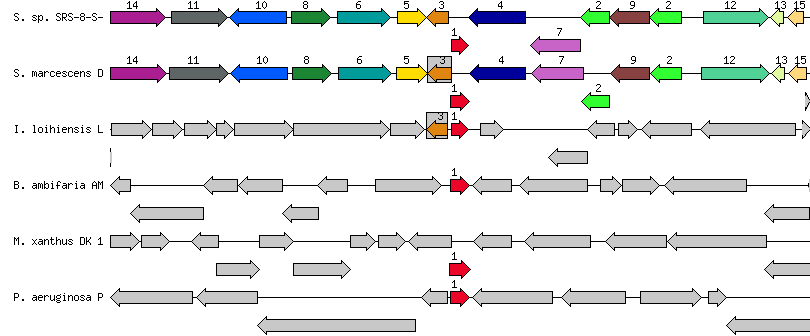


**Fig. SI-2A**

*Pseudomonas aeruginosa* PAO1

*Burkholderia ambifaria AMMD*

*Serratia marcescens* Db11

*Serratia* sp. strain SRS-8-S-2018

*Myxococcus xanthus* DK 1622

*Idiomarina loihiensis* L2TR


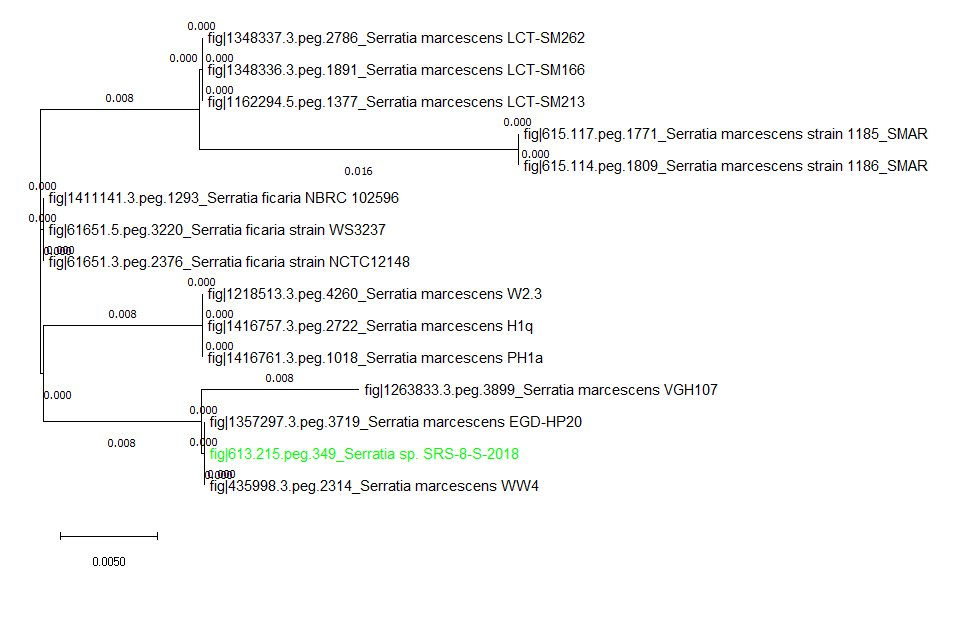


**Fig. SI-2B**

**Fig. SI-2.** Shown is the genomic organization of the transcriptional regulator, MerR family in *Serratia* sp. SRS-8-S-2018 relative to other bacteria (A). The chromosomal region of the focus gene (top) is compared with 5 other organisms with highest similarity to the genera shown. The graphic is centered on the focus gene, which is red and numbered 1. Sets of genes with similar sequence are grouped with the same number and color. Genes whose relative position is conserved in at other species are functionally coupled and share gray background boxes. The focus gene points to the right, even if it is located on the minus strand; B, amino acid based phylotree of the MerR gene using PATRIC and drawn created using the neighbor-joining method in MEGA-X.
